# AKT1-CREB stimulation of PDGFRα expression is pivotal for PTEN deficient tumor development

**DOI:** 10.1038/s41419-021-03433-0

**Published:** 2021-02-10

**Authors:** Xiaofeng Wan, Meng Zhou, Fuqiang Huang, Na Zhao, Xu Chen, Yuncui Wu, Wanhui Zhu, Zhaofei Ni, Fuquan Jin, Yani Wang, Zhongdong Hu, Xianguo Chen, Min Ren, Hongbing Zhang, Xiaojun Zha

**Affiliations:** 1 Department of Biochemistry & Molecular Biology, School of Basic Medicine, Anhui Medical University, Hefei, China; 2 Department of Laboratory, Cancer Hospital, Chinese Academy of Sciences, Hefei, China; 3 State Key Laboratory of Medical Molecular Biology, Department of Physiology, Institute of Basic Medical Sciences, Chinese Academy of Medical Sciences & Peking Union Medical College, Beijing, China; 4 Department of Breast Surgery, The First Affiliated Hospital of Anhui Medical University, Hefei, China; 5 Modern Research Center for Traditional Chinese Medicine, School of Chinese Materia Medica, Beijing University of Chinese Medicine, Beijing, China; 6 Department of Urology, The First Affiliated Hospital of Anhui Medical University, Hefei, China

**Keywords:** Oncogenes, Tumour-suppressor proteins

## Abstract

As evidenced by the behavior of loss-of-function mutants of PTEN in the context of a gain-of-function mutation of AKT1, the PTEN-AKT1 signaling pathway plays a critical role in human cancers. In this study, we demonstrated that a deficiency in PTEN or activation of AKT1 potentiated the expression of platelet-derived growth factor receptor α (PDGFRα) based on studies on Pten−/− mouse embryonic fibroblasts, human cancer cell lines, the hepatic tissues of Pten conditional knockout mice, and human cancer tissues. Loss of PTEN enhanced PDGFRα expression via activation of the AKT1-CREB signaling cascade. CREB transactivated PDGFRα expression by direct binding of the promoter of the PDGFRα gene. Depletion of PDGFRα attenuated the tumorigenicity of Pten-null cells in nude mice. Moreover, the PI3K-AKT signaling pathway has been shown to positively correlate with PDGFRα expression in multiple cancers. Augmented PDGFRα was associated with poor survival of cancer patients. Lastly, combination treatment with the AKT inhibitor MK-2206 and the PDGFR inhibitor CP-673451 displayed synergistic anti-tumor effects. Therefore, activation of the AKT1-CREB-PDGFRα signaling pathway contributes to the tumor growth induced by PTEN deficiency and should be targeted for cancer treatment.

## Introduction

Phosphatase and tensin homologue deleted on chromosome 10 (PTEN) is the second most mutated tumor suppressor gene in human cancers^1^. PTEN acts mainly as a negative regulator of the phosphoinositide 3-kinase (PI3K) pathway by means of its lipid phosphatase activity^2^. PTEN opposes PI3K through dephosphorylation of phosphatidylinositol-3,4,5-trisphosphate(PIP_3_) to phosphatidylinositol-4,5-trisphosphate (PIP_2_)^2^. Upon PTEN loss, PIP3 accumulates and promotes the recruitment of a subset of molecules containing a pleckstrin-homology (PH) domain to cellular membranes, such as the serine/threonine kinases AKT and PDK1^1^. Once positioned at cell membranes, AKT is activated via phosphorylation at Thr^308^ by PDK1 and Ser^473^ by the mTOR kinase complex 2 (mTORC2)^1^. The serine/threonine kinase AKT/PKB family comprises AKT1, AKT2, and AKT3^3^. AKT family members exhibit distinct physiology and pathological roles in humans. The loss-of-function of AKT2 has been shown to contribute to type 2 diabetes^4,5^, while the gain-of-function of AKT2 can promote hypoglycemia syndrome^6^. Moreover, ATK3 plays an important role in brain development^7^. Unlike AKT2 and AKT3, dysregulation or activation of AKT1 is essential for tumor development. Activated AKT1 phosphorylates a wide spectrum of substrates, leading to the promotion of cell survival, cell growth, cell migration, angiogenesis, protein synthesis, and glucose metabolism^2^. However, the underlying molecular mechanism by which the PTEN-AKT1 pathway promotes tumorigenesis remains unclear.

Platelet-derived growth factor (PDGF) stimulates a variety of cell processes including cell proliferation, survival, angiogenesis, and differentiation through binding to α and β tyrosine kinase receptors, PDGFRα, or PDGFRβ^8^. Upon PDGF binding, dimerization of α and β subunits occurs, leading to activation of the intrinsic tyrosine kinase activity of these receptors, and then activates a series of downstream signal transduction pathways^9^. Accumulating evidence indicates that PDGF-related signaling plays an important role in the development of many diseases, including atherosclerosis, fibrosis, and cancers^10–12^. Intriguingly, PDGFRα and PDGFRβ exhibit distinct effects in the pathogenesis of many of these diseases^12,13^. We have previously reported that hyperactivated mammalian target of rapamycin (mTOR) leads to the downregulation of PDGFRβ in Pten-null mouse embryonic fibroblasts (MEFs)^11^. However, the significance of PDGFRα in a Pten deficiency context remains largely elusive.

In this study, we found that PDGFRα was significantly upregulated in Pten−/− MEFs and the hepatic tissues of Pten conditional knockout mice, as well as human cancer cells and tissues. Moreover, the high expression of PDGFRα was associated with poor prognosis in multiple tumors. Further experiments indicated that loss of PTEN led to the upregulation of PDGFRα through activation of the AKT1-cAMP-response element-binding protein (CREB) pathway, and PDGFRα overexpression contributed to the cell proliferation and tumoral growth caused by PTEN deficiency. Also, we demonstrated that a combination of the AKT-specific inhibitor MK-2206 and the PDGFR-specific inhibitor CP-673451 may be exploited as a novel regimen for the treatment of PTEN deficiency-related tumors.

## Materials and methods

### Reagents, plasmids, and antibodies

MK-2206, LY294002, BAY 11-7082, and CP-673451 were purchased from Selleck Chemicals (Houston, TX, USA). 666-15 was obtained from Tocris Bioscience (Bristol, UK). Puromycin was purchased from Solarbio (Beijing, China). Mouse PDGFA factor from Novus Biologicals (Colorado, USA). pLXIN-mutAKT1 (AKT1E17K), pLXIN-myrAKT1 (myristoylated AKT1) expression plasmids, and the empty control pLXIN vector have been described previously^14^. The CREB S133A cDNA was amplified using pCF-CREB M1 plasmid (#22969, Addgene, Watertown, MA, USA) as templates and subcloned into a pLXIN retroviral vector. pGL3-Basic and pRL-TK plasmid were from Promega (Madison, WI, USA). PTEN (#9559), PDGFRα (#3174), PDGFRβ (#4564), p-AKT (Ser-473) (#4060), AKT1 (#2967), p-AKT1 (Ser-473) (#9018), CREB (#9197), p-CREB (Ser133) (#9198), p-PDGFRα^Y849^/PDGFRβ^Y857^ (#3170), cleaved caspase-3 (#9664), AKT2 (#3063), AKT3 (#3788), p-IκBα (#2859), IκBα (#4814), FOXO1 (#2880), FOXO3a (#2497), LaminB1 (#13435), Ki-67 (#12202), GAPDH (#2118) and β-actin (#4970) antibodies were from Cell Signaling Technology (Danvers, MA, USA). All horseradish peroxidase (HRP)-labeled secondary antibodies were from Jackson Immuno Research (West Grove, PA, USA).

### Cell cultures and clinical tissue samples

Pten+/+ and Pten−/− mouse embryonic fibroblasts (MEFs) have been described previously^14,15^. HEK293T, MDA-MB-231, and OVCAR3 cells were obtained from the American Type Culture Collection (Manassas, VA, USA). U373 cells were from GeneChem (Shanghai, China). PT67 cells were from Clontech (Mountain View, CA, USA). HGC-27 cells were from the Baioujing Biotechnology Co., LTD (Shanghai, China). All cells were maintained and propagated in Dulbecco’s modified eagle’s medium with 10% fetal bovine serum and 1% penicillin/streptomycin in a humidified atmosphere of 5% CO_2_ at 37 °C. Mycoplasma test was performed using TaKaRa PCR Mycoplasma Detection Set (Clontech Laboratories, EUA), before all experiments. For in vitro assays, the drug concentration was chosen as suggested by the manufacturer’s protocols or according to previous studies^16–20^. Clinical samples of invasive breast cancer and their paired normal tissues (adjacent normal tissues) were obtained from patients undergoing surgical resection in the Department of Breast Surgery, the First Affiliated Hospital of Anhui Medical University from March 2017 to June 2019. After surgery, all samples were immediately frozen in liquid nitrogen for storage at −80 °C cryogenic refrigerator until analysis. The tissue samples were collected and used after obtaining approval from the Ethics Committees of the First Affiliated Hospital of Anhui Medical University. Written informed consent was obtained from all of the patients who participated in this study according to the committee’s regulations.

### Cell fractionation and Western blotting

The nuclear protein fraction was extracted using a NE-PER Reagent Kit (Pierce) according to the manufacturer’s instructions. Cell or tumor tissue lysates were separated by NuPAGE 10% or 4–12% Bis–Tris Gels (Life Technologies, Carlsbad, CA, USA) and then transferred to PVDF membrane (Millipore, Billerica, MA, USA). The membranes were blocked with 5% nonfat milk in 1 × Tris buffered saline with 0.1% Tween-20 (TBST) for 1 h at room temperature, followed by incubated with the appropriate primary antibody overnight at 4 °C. After washing with TBST, the membranes were incubated with HRP-labeled secondary antibodies and then detected by chemiluminescence.

### Quantitative real-time PCR (qRT-PCR)

Total RNA was extracted from cells using TRIzol (Life Technologies) according to the protocol provided by the manufacturer. RNA was reverse-transcribed to cDNA using the PrimeScript^TM^ RT Reagent Kit (TaKaRa, Shiga, Japan). After 5-fold dilution, 1 μl of cDNA was used as the template in a quantitative real-time PCR reaction. qRT-PCR was performed using Power SYBR® Master Mix (Life Technologies) according to the manufacturer’s protocol. Oligonucleotide primers were synthesized to detect PDGFRα with β-actin as an internal control. The primer sequences were as follows: mouse PDGFRα forward, 5′-TCCATGCTAGACTCAGAAGTCA-3′ and reverse 5′-TCCCGGTGGACACAATTTTTC-3′; β-actin forward, 5′-AGAGGGAAATAGTGCGTGAC-3′ and reverse 5′-CAATAGTGATGACCTGGCCGT-3′.

### Immunofluorescence assays

Treated cells were harvested using trypsin and seeded on slides the day before immunofluorescence assays. The next day, cells were fixed with 4% formaldehyde, followed by the treatment with 1% Triton X-100 (Sigma, St. Louis, MO, USA) for permeabilization and then blocked with 2% bovine serum albumin for 40 min at room temperature. To visualize p-CREB, coverslips were incubated with an anti-p-CREB antibody overnight and then incubated with a FITC-conjugated secondary antibody (Cell Signaling Technology) for 1 h. DAPI (Sigma) was included during staining to visualize the nuclei of cells. Images were captured using a LSM880 + Airyscan confocal laser scanning microscope (Carl Zeiss, Oberkochen, Germany) equipped with a 20X objective (NA 0.8). The fluorescence intensity was analyzed using ImageJ software (National Institutes of Health, Bethesda, MD, USA) based on previously described procedures^21^.

### RNA interference

Cells seeded into 12-well plates were transfected with siRNAs using siRNA-Mate (GenePharma, Shanghai, China) according to the manufacturer’s protocol. Cell lysates were collected 48 h after transfection for further analysis by western blot or qRT-PCR as described above. siRNAs targeting human AKT1 (sc-29195) were obtained from Santa Cruz Biotechnology. All the other siRNA oligonucleotides were purchased from GenePharma. The siRNA target sequences used are as follows: AKT1-1 (mouse), 5′-CAGGCGAUGUACAAACAUA-3′; AKT1-2 (mouse), 5′-GAGCGGGAGGAGUGGACAA-3′; AKT1 (human), 5′-AGGAAGUCAUCGUGGCCAA-3′; AKT2-1 (mouse), 5′-CGCCAUGGAUUACAAGUGU-3′; AKT2-2 (mouse), 5′-CCAUGAAUGACUUCGAUUA-3′; AKT3-1 (mouse), 5′-AAGAGGGUUGGGUUCAGAAGA-3′; AKT3-2 (mouse), 5′-AAGGAUGAAGUGGCACACACU-3′; CREB-1 (mouse), 5′-GUCUCCACAAGUCCAAACA-3′; CREB-2 (mouse), 5′-CAGGCGAUGUACAAACAUA-3′; CREB-1 (human), 5′-GGUGGAAAAUGGACUGGCU-3′; CREB-2 (human), 5′-GAGAGAGGUCCGUCUAAUG-3′; Negative control (NC), 5′-UUCUCCGAAGGUGUCACGU-3′.

### Mouse liver assessment

The liver tissues were collected from Pten^fl/fl^ and Alb-Cre^+^ Pten^fl/fl^ mice by the age of 4 months. All of the mice used for this study are male unless as specified. Albumin-Cre (stock no. 003574) and Pten^fl/fl^ (stock no. 006068) mice were from Jackson Laboratory (Bar Harbor, USA). Alb-Cre mice were crossed with Pten^fl/fl^ mice to generate Alb-Cre Pten^fl/+^ mice, which were then back-crossed with Pten^fl/fl^ mice to obtain liver-specific Pten knockout Alb-Cre^+^ Pten^fl/fl^ mice. All mice were C57BL/6 background and maintained in a pathogen-free facility under a 12-h light/12-h dark cycle with appropriate temperature and humidity. The animal experiments were approved by the Animal Care and Use Committee of Peking Union Medical College and performed in accordance with international guidelines.

### Reporter constructs and luciferase reporter assay

A 1599-bp fragment of the mouse PDGFRα promoter (−1552/+46) was obtained by PCR using mouse genomic DNA extracted from Pten−/− MEFs. Primer sequences used were as follows: forward, 5′-GGGGTACCCCACATTTCGCCTTCAACGG-3′; reverse, 5′-CCCTCGAGGGCAACAGTAATGGGCTCAAA-3′. The amplified DNA fragments were cloned into the pGL3-Basic plasmid through the *Kpn* I and *Xho* I sites. The potential CREB binding site on the promoter of the mouse PDGFRα gene was mutated using the Q5 site-directed mutagenesis kit (NEB, Ipswich, MA, USA). The primer sequences were as follows: PDGFRα_mut_, forward, 5′-TTGGTAGTCGATACAAGCCTGAGCGTTT-3′; reverse, 5′-AAACGCTCAGGCTTGTATCGACTACCAA-3′. For luciferase reporter assays, cells were cultured in triplicate to 80% confluence in 24-well plates and co-transfected with the promoter constructs (200 ng) and the internal control plasmid pRL-TK (20 ng). Luciferase activity was detected with the Dual-Luciferase Reporter Assay System (Promega).

### Chromatin immunoprecipitation (ChIP)

ChIP assay with an anti-p-CREB antibody (#9198, Cell Signaling Technology) to detect protein-DNA interactions was performed using a SimpleChIP® Plus Enzymatic Chromatin IP kit (Cell Signaling Technology) according to the manufacturer’s protocol. The immunoprecipitated DNA was purified and analyzed by qRT-PCR. The primer sequences were as follows: the putative CREB-binding site region (PBR) of mouse PDGFRα, forward: 5′-GCAGAGGGCAGGCATTTGGTAGTC-3′; reverse: 5′-CGACCTTTATCCCTTCGGAGCCAC-3′; a nonspecific CREB-binding region (NBR) of mouse PDGFRα, forward: 5′-CCAACTCCACTGTTTATTGCCCCG-3′; reverse: 5′-CGCGCCCAGGAAGAAAGTAGAAGC-3′.

### Virus production and cell transduction

GV367 lentiviral plasmid expressing mouse PDGFRα, human PTEN, and the empty vector were purchased from GeneChem. The GV118 lentiviral shRNA expression vectors targeting PDGFRα and the control scrambled shRNA (shSc) were obtained from GenePharma. The target sequences were as follows: shPDGFRα (mouse), 5′-CCTGGAGAAGTGAGAAACA-3′; shPDGFRα (human), 5′-GCTTGAAGGCAGGCACATT-3′; shSc, 5′-TTCTCCGAACGTGTCACGT-3′. Lentiviruses were generated by co-transfecting a recombinant vector or a control vector with the packaging vectors (pVSVG, pREV, and pMDL) into HEK293T cells. 48–72 h after infection, culture supernatants were collected and filtered, and then used to infect target cells with a multiplicity of infection (MOI) of 20–50.

Production of retroviruses and subsequent generation of stable gene expression cell lines have been described previously^14^. In brief, the recombinant plasmids (pLXIN-mutAKT1, pLXIN-myrAKT1, or pLXIN-CREB S133A) or the empty vector pLXIN were transfected into the retroviral packaging cell line PT67 using Lipofectamine 3000 (Life Technologies). After 48 h of transfection, the transduced cells were selected with 5 μg/ml hygromycin for stably expressing cells. Conditioned culture supernatants containing viruses were harvested and then filtered through a 0.45-μm filter for the transduction of target cells.

### Cell proliferation and viability assay

Cell proliferation was measured using MTT assay as described previously^22^. In brief, cells were seeded in 96-well plates at 1500 cells/well. After incubated for 8 h, the proliferation was monitored for up to 3 days according to the manufacturer’s specifications. For the cell viability assays, Pten−/− MEFs, Pten+/+ MEFs were seeded in triplicate in 96-well plates at a density of 3000 cells/well and treated with MK-2206, CP-673451, or a combination of MK-2206 and CP-673451 for 48 h. After that, added 5 mg/mL MTT solution (Beyotime, Haimen, China) to each well and incubated the plates for 2 h. Followed by dissolving blue formazan crystal in 100 μl DMSO, the spectrometric absorbance at 570 nm was determined with a microplate reader (Thermo Scientific, Waltham, MA, USA).

### Colony formation assay

Cells were seeded into 100 mm cell culture dish at a density of 500 cells per dish. After incubation for approximately 10 days in DMEM containing 10% FBS, the cells were fixed with methanol and stained with 0.1% crystal violet (1 mg/ml). The number of colonies containing over 50 cells was counted.

### Induction of subcutaneous tumors and combination treatment with MK-2206 and CP-673451 in nude mice

Subcutaneous tumors were established in nude mice as described previously^23^. Immunodeficient BALB/c nude male mice (16–18 g, 5 weeks) were purchased from Vital River Laboratories Animal Technology (Beijing, China). Five nude mice were used in each cohort. Tumor growth was assessed over a 30-day period following subcutaneous inoculation of 5 × 10^6^ Pten−/− MEFs expressing shPDGFRα or shSc in 0.2 ml of DMEM in the right anterior armpit.

To evaluate the therapeutic efficacy of MK-2206 and CP-673451 in vivo, U373 cells (1 × 10^7^ cells/injection) were injected subcutaneously into the right posterior hind region of each nude mouse. After the tumors were detectable, the mice were randomly divided into four groups (each group contained five mice) and treated with either vehicle solution alone (75% DMSO and 25% PBS), MK-2206 (60 mg/kg), CP-673451 (20 mg/kg), or a combination of MK-2206 and CP-673451 by intraperitoneal injection, respectively (I.P., every two days, for a total of 10 injections). Dose selection was based on related literature and preliminary experiments^18,24^. In our preliminary experiments, the mice were randomly divided into seven groups (each group contained three mice), we used MK-2206 at 20, 60, and 120 mg/kg and CP-673451 at 5, 20, and 60 mg/kg to treat tumor-bearing mice. According to the tumor inhibition rate and the health of these nude mice, we chose 60 mg/kg for MK-2206 and 20 mg/kg for CP-673451 in this combined study. Both drugs at a dose of 60 mg/kg of MK-2206 or 20 mg/kg of CP-673451 led to a medium suppression of tumor growth without influencing the body weight of mice (Supplementary Fig. S1). Tumor dimensions were measured with a digital caliper every two days, and the tumor volume was calculated using the formula: *V* = 1/2 (width^2^ × length). Body weights were also monitored. The mice were euthanized on day 35 after inoculation with cancer cells for subsequent analysis. All animals were maintained and used in strict accordance with the guidelines of the Animal Center of Anhui Medical University, and all animal experimental procedures were approved by the Experimental Animal Ethical Committee of Anhui Medical University.

### Immunohistochemistry (IHC) analysis

Immunohistochemical analysis was performed as described previously^23^. In brief, tumor tissues were fixed in 4% paraformaldehyde and embedded in paraffin. Sections (4 μm) were prepared for indicated primary antibodies or hematoxylin and eosin (H&E) staining according to standard protocols.

### Generation of Pten knockout cell lines using CRISPR-Cas9

Pten knockout MDA-MB-231 or OVCAR3 cell lines were generated following a protocol from the Feng Zhang lab (https://zhanglab.bio). sgRNAs (forward: CACCGGCATATTTATTACATCG; reverse: AAACCGATGTAATAAATATGCC) targeting the genomic sequence of Pten were cloned into the Lenti-CRISPRv2 vector (Addgene, #52961). For virus production, these recombinant plasmids were transfected into HEK 293 T cells with packing vectors (psPAX2 and pVSVG). Cell supernatant was harvested and filtered before being used for infection of target cells, followed by selection with puromycin (Sigma-Aldrich) for 2 weeks. Monoclonal cell lines were thus generated, and the knockout of PTEN was verified by Western blot. An empty Lenti-CRISPRv2 vector was used to generate a control cell line.

### Apoptosis assays

Cell apoptosis was measured using an Annexin V-FITC apoptosis kit (BD Pharmingen, San Diego, CA, USA) according to the manufacturer’s instructions. Briefly, the cells were harvested after treatment, washed twice with PBS, and resuspended in 1 × binding buffer. Annexin V-FITC and PI (BestBio, Shanghai, China) were added to the cell preparations, and these preparations were then incubated for 15 min in the dark. Cell apoptosis was monitored by Annexin V staining on a FACS Calibur flow cytometer (Becton Dickinson, San Diego, CA, USA).

### Gene set enrichment analysis

RNA sequencing data from breast cancer (*n* = 1085), ovarian cancer (*n* = 426), gastric cancer (*n* = 408), and bladder cancer (*n* = 404) patients were obtained from The Cancer Genome Atlas (TCGA) (http://cancergenome.nih.gov/). Gene set enrichment analysis was performed to examine the enrichment of PI3K/AKT positively regulated gene sets in PDGFRα-high and PDGFRα-low breast cancer, ovarian cancer, gastric cancer, and bladder cancer tissues, according to methods described previously^25^.

### Statistics

All data were found in a normal distribution, and variance was similar among the groups statistically compared. No samples or mice were excluded from the analysis as outliers. Investigators were not blinded to the group allocation during the experiment and out-come assessment. The sample sizes of all experiments were selected based on our experience and previous studies^23,26^. The difference between two groups was evaluated using a two-tailed Student’s *t* test. One-way ANOVA was used to evaluate differences for multiple comparisons. A Kaplan–Meier survival analysis and logarithmic rank test were used to compare the survival of patients. Data are presented as the mean ± SD from at least triplicate samples. All statistical analyses were performed using the GraphPad Prism 6.0 software. Differences were considered significant when *P* < 0.05.

## Results

### Loss of PTEN upregulated PDGFRα through the activation of AKT1

**Fig. 1:**
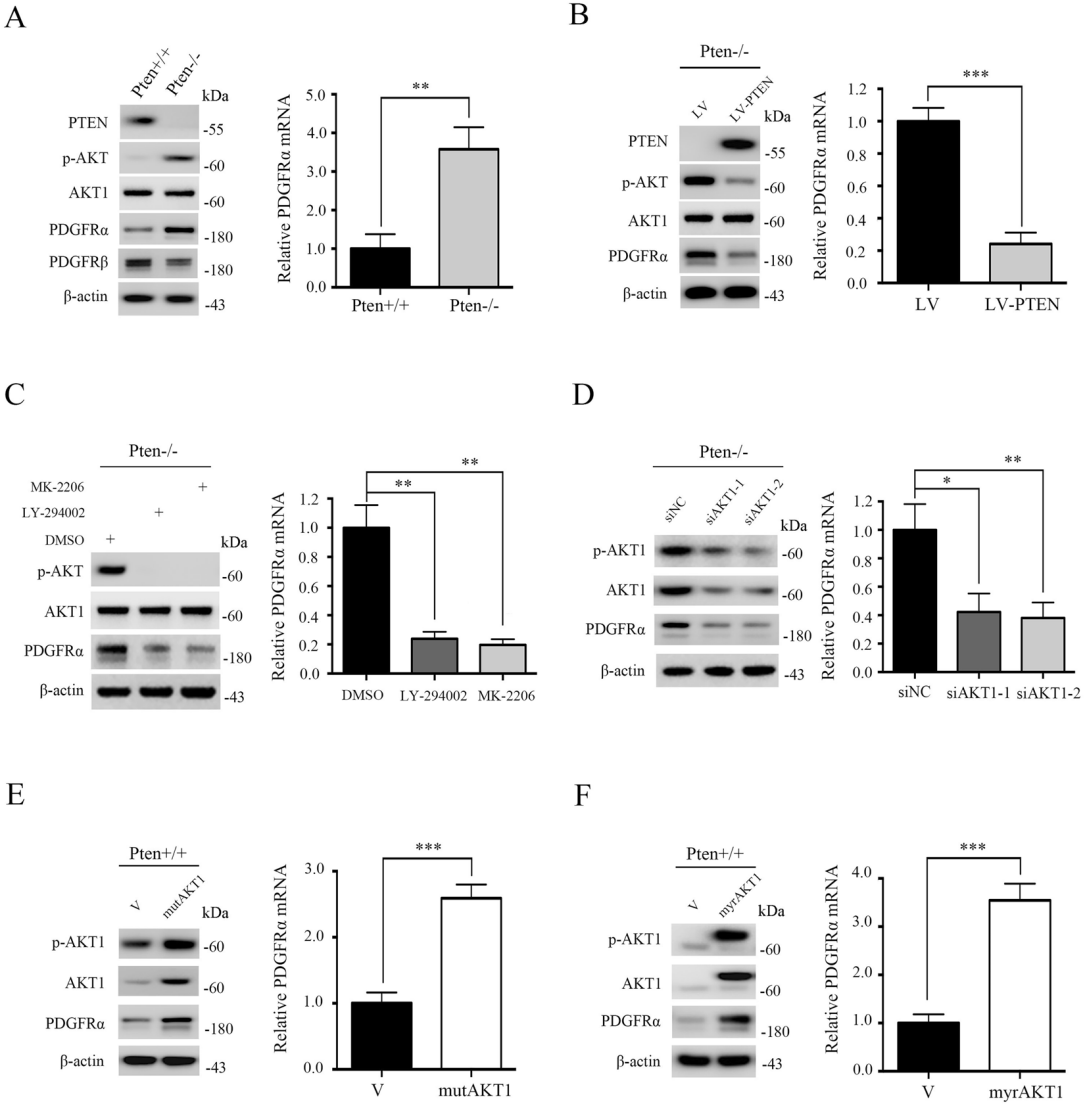
Loss of PTEN upregulated PDGFRα through the activation of AKT1. **A** Pten+/+ and Pten−/− MEFs. **B** Pten−/− MEFs were infected with lentivirus harboring a vector encoding human PTEN (LV-PTEN) or the empty vector (LV). **C** Pten−/− MEFs were treated with LY-294002 (10 μM) or MK-2206 (2 μM) for 24 h. **D** Pten−/− MEFs were transfected with control siRNAs (siNC) or two independent siRNAs against AKT1 (siAKT1-1 and siAKT1-2) for 48 h. **E**, **F** Pten+/+ MEFs transduced with the retroviruses for AKT1E17K (mutAKT1) (**E**) or myristoylated AKT1 (myrAKT1) (**F**) in pLXIN or its control vector pLXIN (V). **A**–**F** Cell lysates were subjected to immunoblotting with the indicated antibodies (left panels). qRT-PCR was performed to detect the mRNA level of PDGFRα (right panels). Error bars indicate mean ± SD of triplicate samples. *P < 0.05; **P < 0.01; ***P < 0.001.

To investigate downstream effectors of PTEN, we analyzed the differences in gene expression profiles between Pten−/− MEFs and Pten+/+ MEFs. Interestingly, unlike PDGFRβ, the mRNA abundance of PDGFRα was significantly elevated in Pten-deficient cells as compared with control cells (Supplementary Table S1). Western blot and qRT-PCR analyses confirmed that PDGFRα expression was dramatically increased in Pten-deficient MEFs (Fig. 1A). Furthermore, the re-introduction of PTEN to Pten−/− MEFs using wild-type human PTEN (hPTEN) cDNA expression lentiviruses significantly blunted the expression of PDGFRα in Pten−/− MEFs (Fig. 1B).

On the premise of PTEN deficiency, the most dominant downstream conduction event is the constitutive activation of AKT^2^. To determine whether AKT was responsible for the upregulation of PDGFRα caused by PTEN deficiency in our MEFs, we treated Pten−/− MEFs with a PI3K inhibitor (LY294002) and a specific AKT inhibitor (MK-2206). Treatment with both of these inhibitors led to a substantial inhibition in the expression of phospho-AKT Ser^473^ and PDGFRα, indicating that there was a functional link between AKT and PDGFRα (Fig. 1C). To explore which one of the 3 AKT isoforms was involved in the regulation of PDGFRα expression, we assessed PDGFRα levels in AKT1-, AKT2-, or AKT3-knockdown Pten−/− MEFs. Knockdown of AKT1 significantly decreased the level of PDGFRα, while knockdown of AKT2 or AKT3 did not alter the expression of PDGFRα (Fig. 1D and Supplementary Fig. S2), suggesting that AKT1 was involved in the regulation of PDGFRα expression. To further verify that activation of AKT1 had an effect on PDGFRα expression, we examined PDGFRα levels in Pten+/+ MEFs ectopically expressing either myristoylated AKT1 (myrAKT1) or an AKT1E17K mutant (mutAKT1). Not surprisingly, the expression of either of these two constitutively active forms of AKT1 both led to a significant elevation of PDGFRα (Fig. 1E, F). Collectively, hyperactivated AKT1 was responsible for the elevated expression of PDGFRα in Pten-deficient cells.

### The PTEN-AKT1 signaling pathway increases PDGFRα expression through the activation of CREB

CREB is a crucial transcription factor and regulates a broad spectrum of biological processes to orchestrate cell growth, differentiation, etc.^27^. The transcriptional activity of CREB is induced upon reversible phosphorylation at various serine residues, in particular at Ser^133^, by various kinases, depending on the cell type and biological context^27^. Currently, CREB has been found to be overexpressed or constitutively phosphorylated in many forms of human cancer^28,29^.

**Fig. 2:**
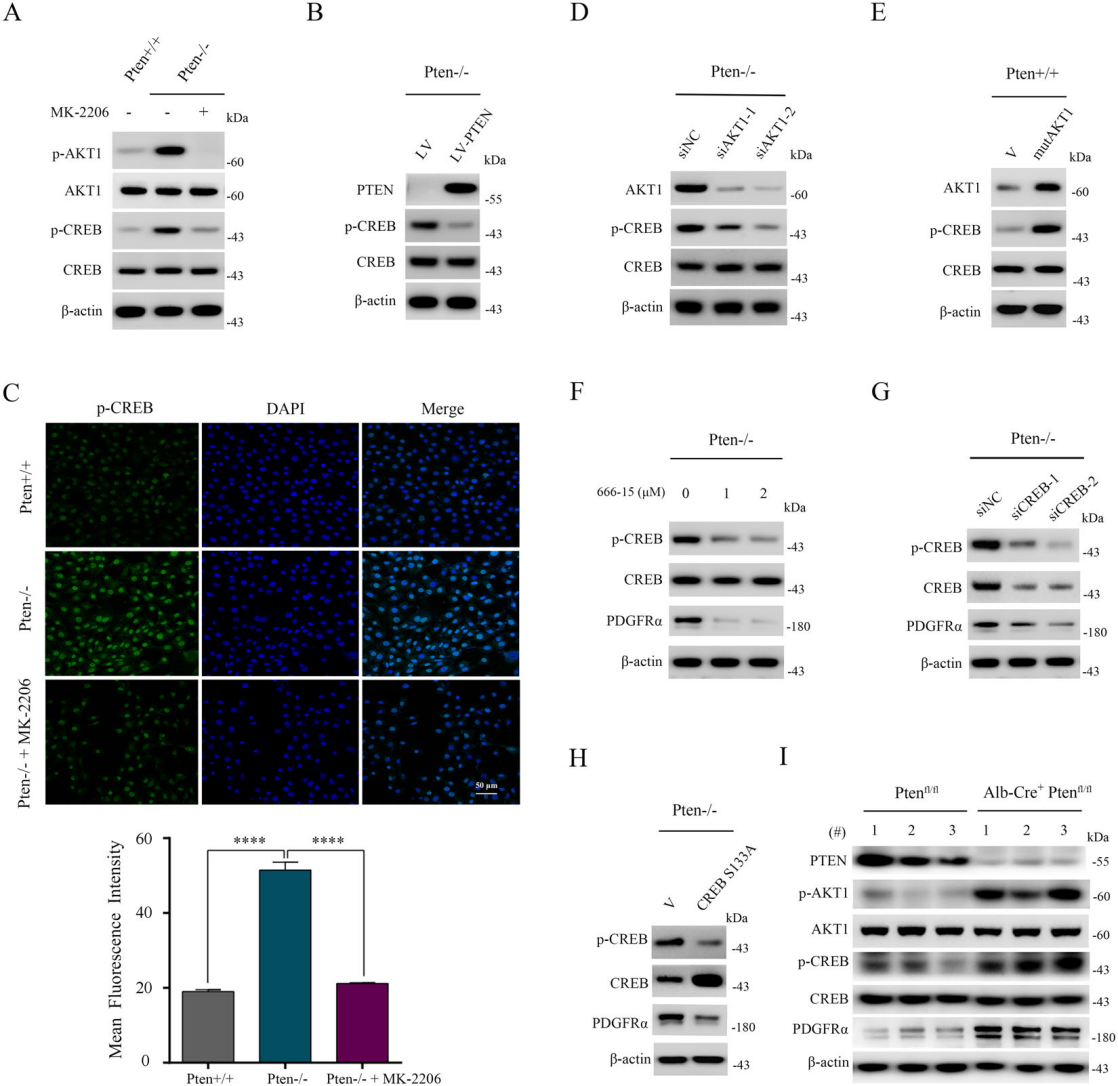
The PTEN-AKT1 signaling pathway increases PDGFRα expression through the activation of CREB. **A**, **C** Pten+/+, Pten−/− MEFs, and MK-2206 (5 μM, 24 h) treated Pten−/− MEFs. **B** Pten−/− MEFs were infected with LV-PTEN or LV lentiviruses. **C** The expression of p-CREB was analyzed by an immunofluorescence assay. Representative images were presented (upper panel).Scale bar, 50 μm. Mean fluorescence intensity was calculated by software ImageJ (lower panel). **D** Pten−/− MEFs were transfected with siNC, siAKT1-1, or siAKT1-2 for 48 h. **E** Pten+/+ MEFs transduced with the retroviruses for AKT1E17K (mutAKT1) in pLXIN or its control vector pLXIN (V). **F** Pten −/− MEFs were treated with or without 666-15 (1 or 2 μM) for 24 h. **G** Pten−/− MEFs were transfected with siNC or two independent siRNAs against CREB (siCREB-1 and siCREB-2) for 48 h. **H** Pten−/− MEFs transduced with the retroviruses for CREB S133A in pLXIN or its control vector pLXIN (V). **I** The whole liver lysates from liver-specific Pten knockout mice (Alb-Cre^+^ Ptenfl/fl) and the control mice (Ptenfl/fl). *n* = 3. **A**, **B**, **and D**–**I** Cell or tissue lysates were subjected to immunoblotting with the indicated antibodies. Error bars indicate mean ± SD of triplicate samples. ****P < 0.0001.

Thus, we tested whether CREB activity was stimulated by AKT1 in Pten-null MEFs. As shown in Fig. 2A, the amount of CREB phosphorylated at Ser^133^ (p-CREB) was dramatically increased in cells lacking PTEN compared with control cells, while its expression was significantly decreased in response to MK-2206 treatment. The re-introduction of PTEN also led to a marked reduction of p-CREB in Pten−/− MEFs (Fig. 2B). Moreover, immunofluorescence analysis showed that the nuclear level of p-CREB was markedly elevated in Pten−/− MEFs, and its expression was dramatically reduced by MK-2206 treatment (Fig. 2C). Furthermore, knockdown of AKT1 led to a substantial decrease of p-CREB expression in Pten−/− MEFs (Fig. 2D). In contrast, Pten+/+ MEFs with constitutively activated AKT1 showed elevated p-CREB levels (Fig. 2E). These data in aggregate indicated that a loss of PTEN activates CREB through the activation of AKT1.

Due to the activation of AKT1 leading to both an increase in CREB activity and PDGFRα expression, we next determined whether AKT1 upregulation of PDGFRα was mediated by CREB in PTEN-deficient cells. As shown in Fig. 2F, the protein level of PDGFRα was markedly decreased in Pten−/− MEFs in the presence of 666-15, a specific inhibitor of CREB. To further establish the relationship between CREB and PDGFRα, siRNAs for CREB were transfected into Pten−/− MEFs. As depicted in Fig. 2G, treatment with CREB siRNAs led to a significant reduction in the expression of CREB as well as PDGFRα. Similarly, overexpression of a dominant-negative CREB (CREB S133A) significantly decreased the expression of PDGFRα in Pten−/− MEFs (Fig. 2H). Additionally, PDGFRα, in alignment with p-AKT1 and p-CREB, was also significantly increased in the liver tissues from liver-specific PTEN knockout mice (Alb-Cre^+^ Pten^fl/fl^), as compared with that from control mice (Pten^fl/fl^) (Fig. 2I). Taken together, these results illustrated that hyperactivated AKT1 caused by a loss of PTEN positively regulated PDGFRα expression through the activation of CREB.

### CREB promotes the transcription of PDGFRα

**Fig. 3:**
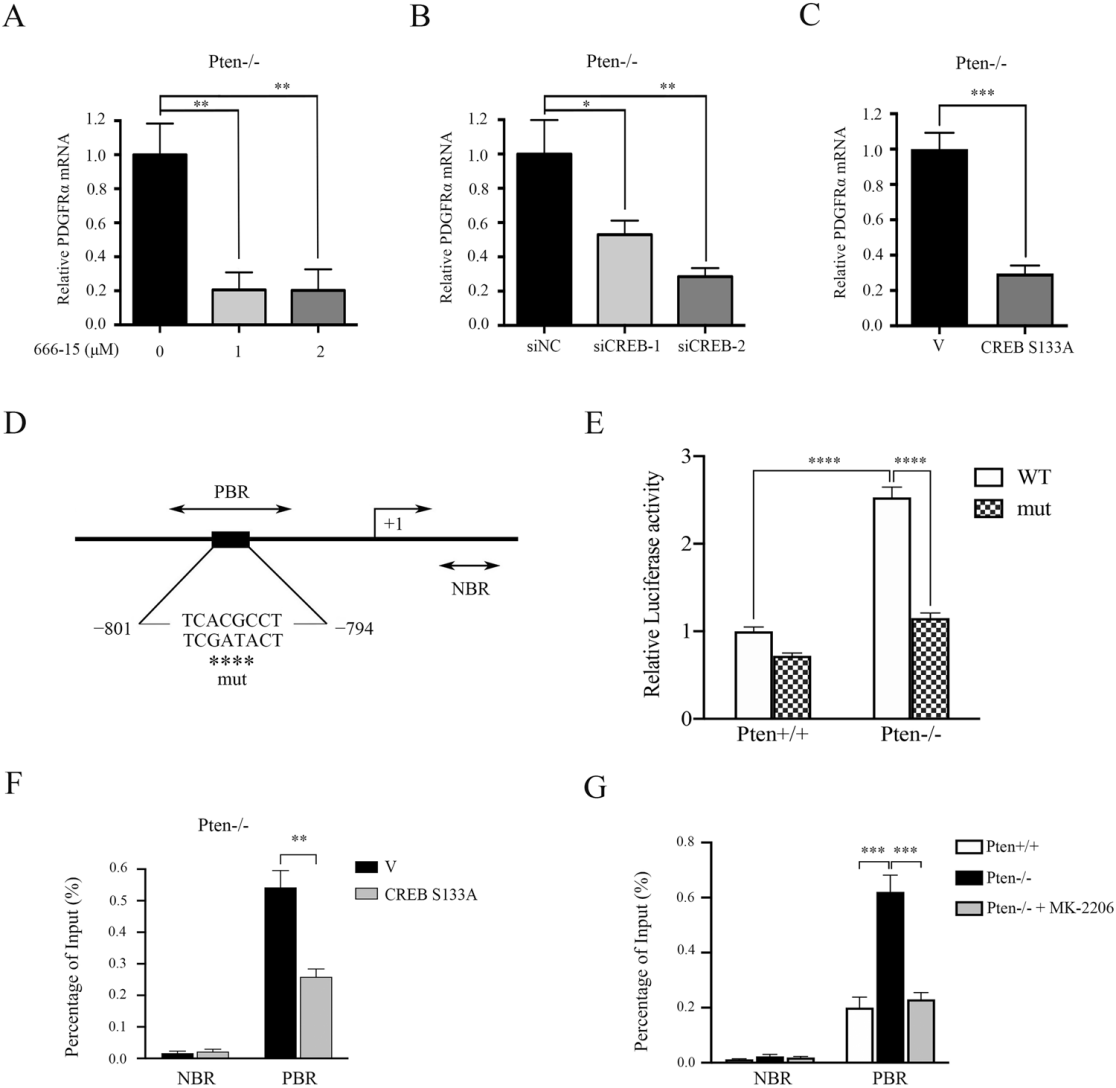
CREB promotes PDGFRα transcription. **A** Pten−/− MEFs were treated with or without 666-15 (1 or 2 μM) for 24 h. **B** Pten−/− MEFs were transfected with siNC, siCREB-1, or siCREB-2 for 48 h. **C**, **F** Pten−/− MEFs transduced with the retroviruses for CREB S133A in pLXIN or its control vector pLXIN (V). **A**–**C** The mRNA level of PDGFRα was detected by qRT-PCR. **D** Schematic representation of the putative wild-type (WT) and mutated (mut) CREB-binding sites in the promoter of the mouse PDGFRα gene. **E** The wild-type (WT) reporter plasmid pPDGFRα-Luc or the mutated (mut) reporter plasmid (pPDGFRαmut-Luc) were co-transfected with Pten+/+ or Pten−/− MEFs with pRL-TK plasmid. Relative luciferase activity was detected 24 h after transfection. **G** Pten+/+, Pten−/− and MK-2206-treated (5 μM, 24 h) Pten−/− MEFs. **F**, **G** The indicated cells were subjected to a ChIP analysis of CREB binding site in PDGFRα promoter region using an anti-phospho-CREB (Ser^133^) antibody. Normal rabbit IgG antibody served as negative control. qRT-PCR was performed to amplify regions surrounding the putative CREB binding region (PBR) and a nonspecific CREB binding region (NBR). The data were plotted as the ratio of immunoprecipitated DNA subtracting nonspecific binding to IgG vs. total input DNA (%). Representative data from three independent experiments are shown. Error bars indicate mean ± SD of triplicate samples. *P < 0.05; **P < 0.01; ***P < 0.001; ****P < 0.0001.

To explore the mechanism involved in increased PDGFRα expression by activation of CREB, total RNA was isolated from 666-15-treated or CREB siRNA-transfected Pten−/− MEFs and their corresponding control cells. qRT-PCR was performed to analyze the mRNA levels of PDGFRα. As shown in Fig. 3A, B, the expression of PDGFRα mRNA was markedly decreased in the presence of either 666-15 or CREB siRNAs in Pten−/− MEFs. Consistently, inhibition of CREB by ectopic expression of CREB S133A resulted in a significantly reduced PDGFRα mRNA level in Pten−/− MEFs, as compared with empty vector transfection control cells (Fig. 3C). These data together suggested that CREB upregulated PDGFRα at the mRNA level.

To further elucidate the underlying mechanisms by which CREB regulates PDGFRα, we analyzed the 5′-flanking sequence of the PDGFRα gene upstream of the transcription start site. A putative CREB binding sequence (−801/−794, TCACGCCT) was identified in the promoter of the PDGFRα gene (Fig. 3D). Next, we cloned the PDGFRα gene promoter (from −1552 to + 46 bp) into a luciferase reporter plasmid for evaluation of promoter activity. This recombinant reporter plasmid was then co-transfected into Pten+/+ and Pten−/− MEFs together with the internal control plasmid pRL-TK. Increased luciferase activity was observed in Pten−/− MEFs as compared with control cells (Fig. 3E). More importantly, this enhanced transcriptional activity was markedly attenuated when the putative CREB binding site was mutated (Fig. 3E). qRT-PCR analysis of ChIP DNA further revealed that the binding of CREB to this putative site was weakened by the overexpression of CREB S133A in Pten−/− MEFs (Fig. 3F). Also, the binding of CREB to this site was drastically stronger in Pten−/− MEFs than in control cells and was attenuated by the suppression of AKT with MK-2206 (Fig. 3G). Taken together, we found that CREB promoted PDGFRα transcription by direct binding to the promoter of the PDGFRα gene in Pten-deficient cells.

### Depletion of PDGFRα suppressed the cell proliferation and tumoral growth of Pten-deficient cells

**Fig. 4:**
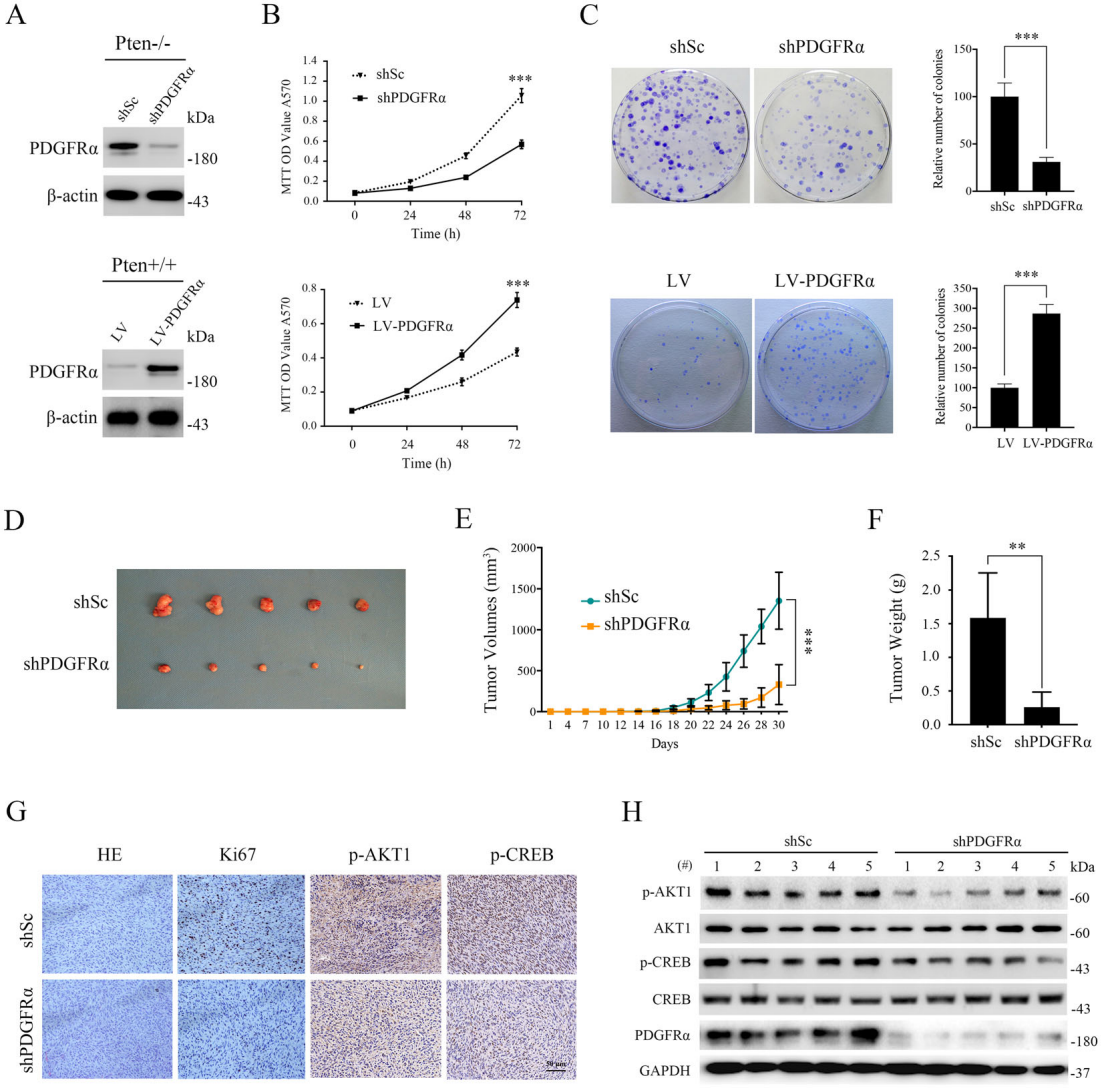
PDGFRα promotes the proliferation of Pten-deficient cells in vitro and in vivo. **A** Pten−/− MEFs were stably expressing shRNAs targeting PDGFRα (shPDGFRα) or a control shRNA (shSc) (upper panel). Pten+/+ MEFs infected with lentivirus harboring a vector encoding PDGFRα (LV-PDGFRα) or the empty vector (LV) (lower panel). Cell lysates were subjected to immunoblotting with the indicated antibodies. **B** Proliferation of the indicated cells was examined using MTT assay. **C** Colonies formed by the indicated cells were stained and counted. Representative images (left panels) and quantifications (right panels). **D**–**H** Pten−/− MEFs infected with shPDGFRα or shSc lentiviruses were inoculated subcutaneously into nude mice, following by monitoring for tumor growth. Tumor pictures (**D**). Tumor volumes at different times (**E**). Tumor weight (**F**). Tumor tissues were embedded with paraffin and then subjected to H&E and immunohistochemistry analysis (200×). Representative images were presented (**G**). Tumor tissue lysates were subjected to immunoblotting with the indicated antibodies. **H** Error bars indicate mean ± SD of triplicate samples. **P < 0.01; ***P < 0.001.

To determine the functional role of PDGFRα in PTEN deficiency-related cell proliferation and tumor growth, we designed additional in vitro and in vivo experiments. First, Pten-null MEFs were infected with lentiviruses for expressing PDGFRα shRNA, which were confirmed by Western blot (Fig. 4A). The knockdown of PDGFRα expression led to a marked reduction in cell proliferation, as demonstrated by MTT and colony formation assays (Fig. 4B, C). Conversely, ectopic expression of PDGFRα significantly accelerated cell proliferation and enhanced colony formation in Pten+/+ MEFs (Fig. 4A–C lower panels).

To investigate the in vivo effect of PDGFRα on the tumoral growth of Pten-deficient cells, we injected Pten-deficient MEFs expressing shPDGFRα or shScramble subcutaneously into the right anterior armpit of nude mice, and tumor growth was monitored for 30 days post-injection. As shown in Fig. 4D–F, depletion of PDGFRα markedly reduced the tumorigenic capacity of Pten−/− MEFs. IHC analysis of Ki-67 expression further confirmed that these tumor tissues with attenuated PDGFRα showed decreased proliferative properties (Fig. 4G). Additionally, reduced PDGFRα expression in tumor tissues derived from Pten−/− MEFs with shPDGFRα was verified by immunoblotting (Fig. 4H). Collectively, these results demonstrated that PDGFRα promoted the cell proliferation and tumoral growth of Pten-deficient cells.

### PTEN regulates the AKT1-CREB-PDGFRα signaling pathway in human cancer cells

**Fig. 5:**
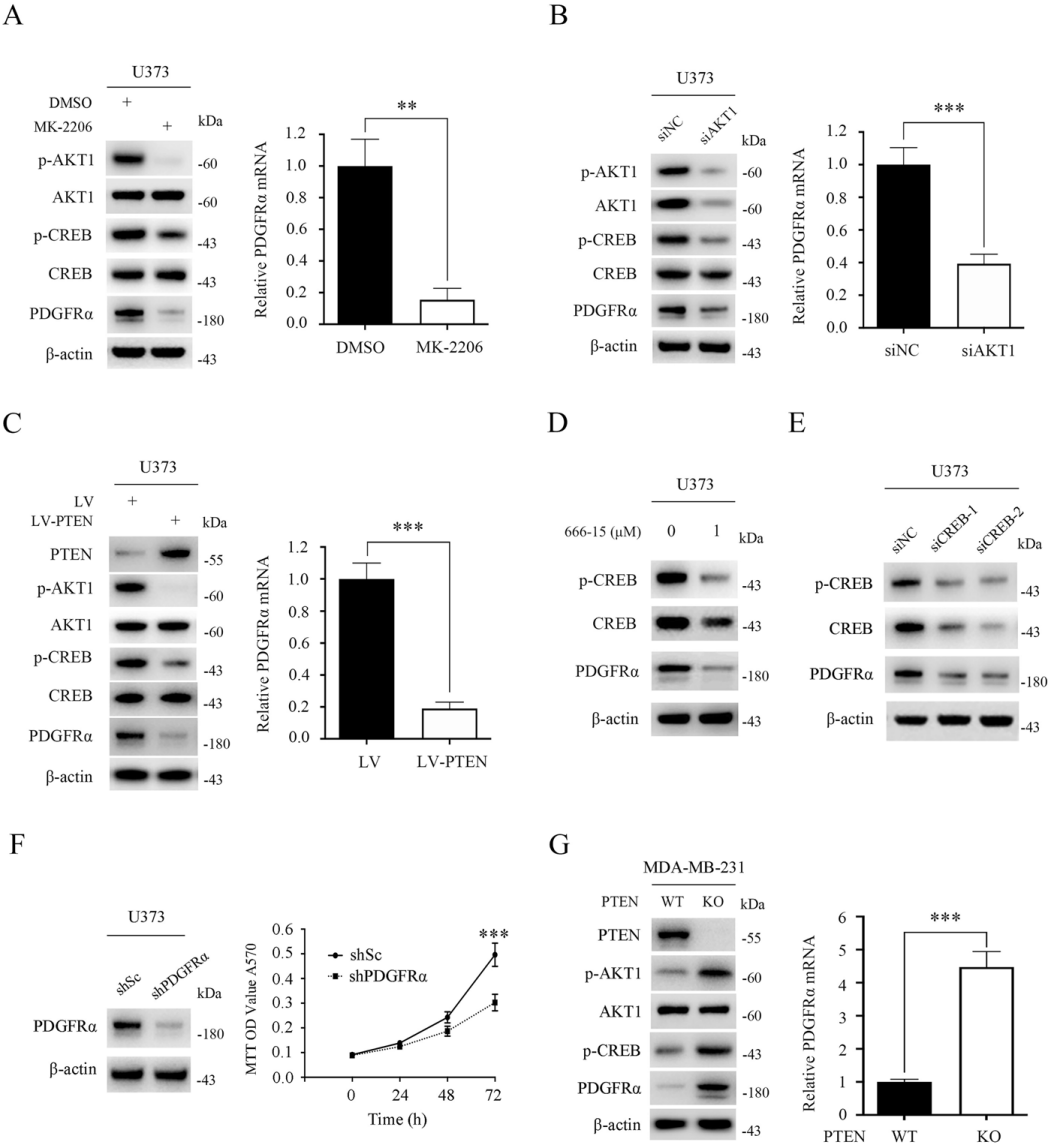
PTEN-AKT1-CREB-PDGFRα signaling pathway exists in human cancer cells. **A** U373 cells were treated with MK-2206 (5 μM) for 24 h. **B** U373 cells were transfected with siNC or siRNA against AKT1 (human siAKT1) for 48 h. **C** U373 cells were transduced with LV-PTEN or LV lentiviruses. **A**–**C** Cell lysates were subjected to immunoblotting (left panels), and qRT-PCR was performed to examine the expression of PDGFRα (right panels). **D** U373 cells were treated with 1 μM 666-15 for 24 h. **E** U373 cells were transfected with siNC or two independent siRNAs against CREB (human siCREB-1 and siCREB-2) for 48 h, respectively. **D**, **E** Cell lysates were subjected to immunoblotting. **F** U373 cells were stably expressing shRNAs targeting PDGFRα (shPDGFRα) or a control shRNA (shSc). Cell lysates were analyzed for PDGFRα expression by western blot (left panel). Proliferation of the indicated cells was examined using an MTT assay (right panel). **G** Pten wild-type (WT) or knockout (KO) MDA-MB-231 cells were subjected to western blot (left panel) and qRT-PCR (right panel) analyses. Error bars indicate mean ± SD of triplicate samples. **P < 0.01; ***P < 0.001.

To evaluate whether this newly discovered PTEN-AKT1 pathway regulation of PDGFRα signaling also occurred in human cancer cells, a PTEN-absent human cancer cell line U373 (a human astrocytoma cell line) was employed. As shown in Fig. 5A, inhibition of AKT1 with MK-2206 led to the downregulation of p-CREB as well as PDGFRα in U373 cells. Consistently, knockdown of AKT1 led to a similar result as MK-2206 treatment (Fig. 5B). Moreover, restoration of PTEN in U373 cells normalized AKT1-CREB-PDGFRα signaling (Fig. 5C). As expected, inhibition of CREB by pharmacological or genetic strategies led to a significant decrease in PDGFRα expression in U373 cells (Fig. 5D, E). Knockdown of PDGFRα significantly suppressed the growth of U373 cells (Fig. 5F). Similar to U373 cells, manipulation of the activity of AKT1-CREB signaling by ectopic expression of PTEN led to decreased expression of PDGFRα in HGC-27 (a Pten-deficient gastric cancer cell line) cells, and depletion of PDGFRα attenuated the proliferation of HGC-27 cells (Supplementary Fig. S3). Additionally, we knocked out the PTEN gene in two other human cancer lines, namely MDA-MB-231 (a human breast cancer cell line) and OVCAR3 (a human ovarian carcinoma cell line) cells. As shown in Fig. 5G and Supplementary Fig. S4, the deletion of PTEN led to the upregulation of p-AKT1 and p-CREB, as well as PDGFRα in both of these two cells. Therefore, the PTEN-AKT1-CREB-PDGFRα signaling cascade was also present in human cancer cells.

### PDGFRα expression correlates with PI3K-AKT signaling in human cancer tissues and associates with poor survival in multiple tumors

**Fig. 6:**
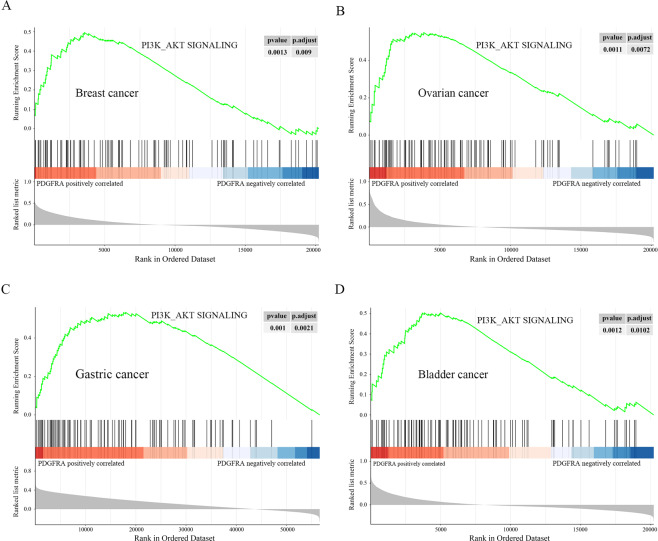
PDGFRα expression positively correlates with PI3K/AKT signaling pathway activity in multiple human cancer tissues. Gene set enrichment analysis (GSEA) comparing the gene sets positively regulated by PI3K/AKT signaling in PDGFRα-high and PDGFRα-low breast cancer (**A**), ovarian cancer (**B**), gastric cancer (**C**), and bladder cancer (**D**) patients, based on The Cancer Genome Atlas (TCGA) RNA-seq datasets. Genes positively correlated with PDGFRα are shown on the left (red zone). Genes negatively correlated with PDGFRα are shown on the right (blue zone).

To investigate the clinical relevance of this newly discovered AKT regulation of PDGFRα in human cancer tissues, we analyzed The Cancer Genome Atlas (TCGA) RNA-seq datasets for a correlation between PDGFRα expression and PI3K-AKT signaling pathway activity in the tumors of breast cancer patients with Gene Set Enrichment Analysis (GSEA). The results showed that the genes positively regulated by PI3K-AKT signaling were enriched in PDGFRα-high expression groups (Fig. 6A). Similarly, our GSEA analysis also demonstrated a positive correlation between PDGFRα expression and PI3K-AKT signaling pathway activity in ovarian cancer, gastric cancer, and bladder cancer (Fig. 6B–D). Therefore, PDGFRα expression positively correlated with PI3K-AKT pathway activity in human cancer tissues.

**Fig. 7:**
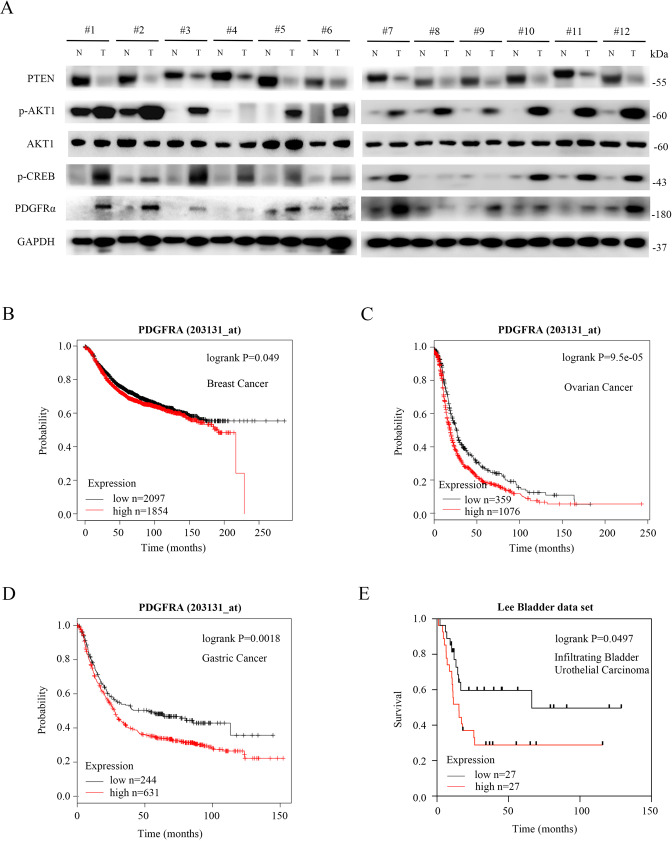
PTEN is negatively correlated with p-AKT1, p-CREB, and PDGFRα in human breast tumors, and PDGFRα is associated with poor survival in multiple tumors. **A** 12 paired of human invasive breast cancer tissues (T) and adjacent normal tissues (N) were subjected to immunoblotting with the indicated antibodies. **B**–**D** Choose the best probe set of PDGFRa (203131_at) in the Kaplan-Meier Plotter database (www.kmplot.com) to analyze the relapse-free survival (RFS) of cancer patients. Patient samples were split into two groups (high vs. low expression) by choosing “Auto select best cutoff”. Draw Kaplan-Meier plot without restrict analysis to any subtypes or clinical cohorts. Survival curve based on overall survival follow-up time as a prognostic indicator in breast cancer (**B**), ovarian cancer (**C**), and gastric cancer (**D**). **E** Analysis of Lee bladder data sets available through Oncomine (www.oncomine.org) indicates a significant correlation between high PDGFRα expression and poor survival in infiltrating bladder urothelial carcinoma. n is the number of patients for each condition.

Next, the expression of PTEN, p-AKT1, p-CREB, and PDGFRα in invasive breast cancer tissues and the corresponding adjacent normal tissues was assessed by Western blot. As depicted in Fig. 7A, p-AKT1, p-CREB, and PDGFRα levels were substantially elevated in almost all of the 12 breast tumor tissues tested, and in which the PTEN levels were significantly decreased as compared with those in corresponding para tumor tissues. Therefore, in addition to Pten-deficient mouse and human cell lines, the regulatory pathway of PDGFRα by the PTEN-AKT1-CREB axis also existed in human tumor tissues.

Furthermore, the clinical significance of PDGFRα was investigated by analyzing survival data from patients with multiple cancers using a Kaplan Meier Plotter (www.kmplot.com) and the Oncomine Database (www.oncomine.org). As shown in Fig. 7B, the high expression of PDGFRα was associated with poor survival in breast cancer. Moreover, the negative correlation between PDGFRα and patient survival was also present in multiple tumors including ovarian cancer, gastric cancer, and bladder cancer (Fig. 7C–E). Together, our results revealed that the PTEN-AKT1-CREB-PDGFRα signaling pathway may exist in human tumors and the high expression of PDGFRα was associated with poor prognosis in tumor patients.

### Combined inhibition of AKT1 and PDGFRα strongly suppressed both in vitro and in vivo proliferation of Pten-deficient cells

**Fig. 8:**
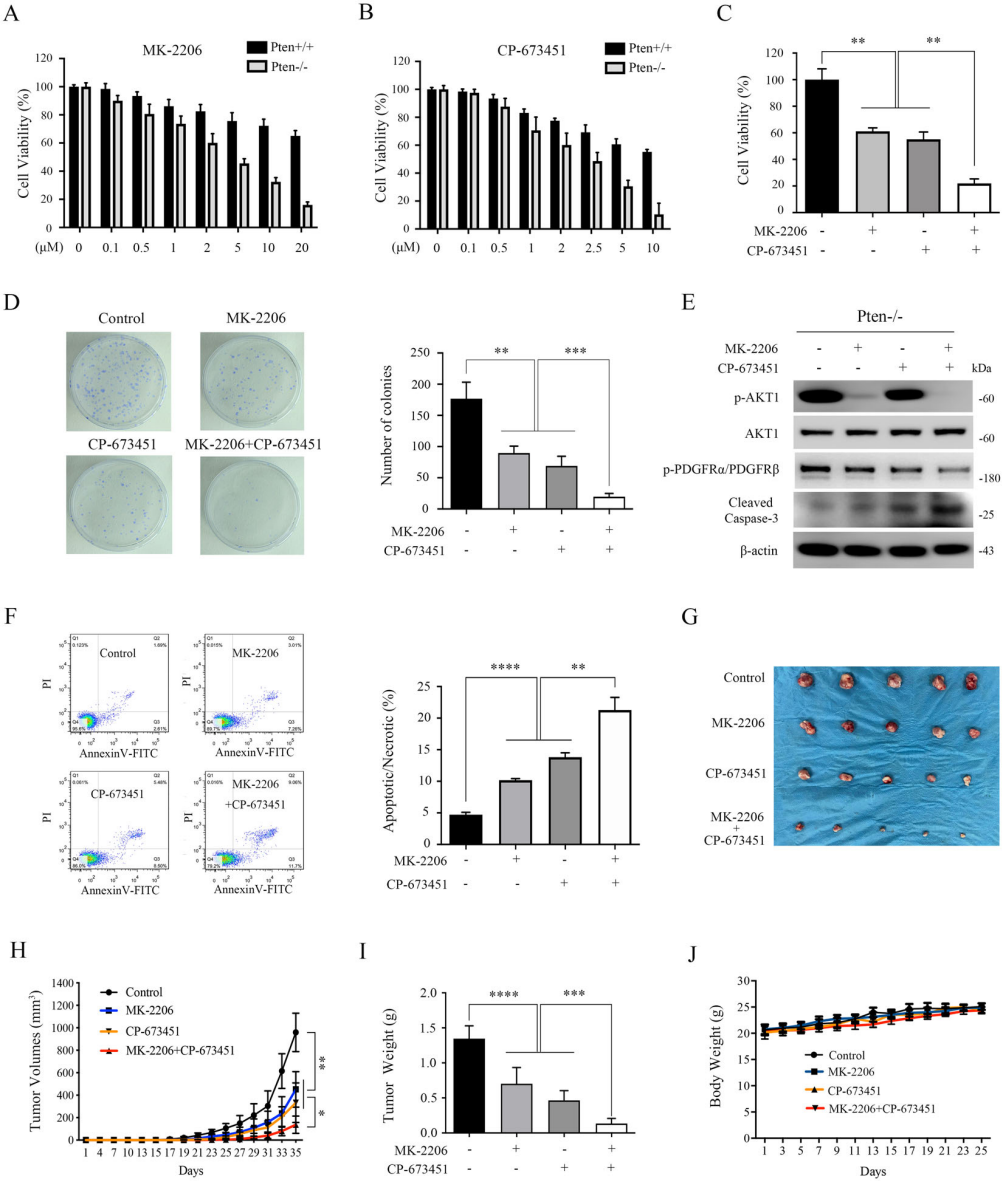
The combination of MK-2206 and CP-673451 exerts a stronger inhibitory effect on the growth of Pten-deficient cells in vitro and in vivo than either agent alone. **A, B** Pten+/+ and Pten−/− MEFs were treated with MK-2206 (**A**) or CP-673451 (**B**) as indicated concentrations for 48 h. Cell viability was measured with an MTT assay. **C**–**F** Pten−/− MEFs were treated with a combination of 2 μM MK-2206 and 2 μM CP-673451 or either agent alone for 48 h. Cell viability was tested by an MTT assay (**C**). The colonies formed by the indicated cells were stained and counted, representative images (left panel) and quantification (right panel) (**D**). Cell lysates were subjected to immunoblotting with the indicated antibodies (**E**). Apoptosis assay was used to detect apoptosis changes in control cells and drug-treated Pten−/− MEFs (**F**). **G**–**J** U373 cells were inoculated subcutaneously into the nude mice to evaluate the effects of MK-2206 or CP-673451, respectively, or in combination in vivo. Dissected tumors (**G**). Tumor volume growth curves (**H**). Tumor weight (**I**). Body weights of mice (**J**). Error bars indicate mean ± SD of triplicate samples. *P < 0.05; **P < 0.01; ***P < 0.001; ****P < 0.0001.

Since PTEN deficiency led to the activation of AKT1 and subsequent significant upregulation of the expression of PDGFRα, Pten-null cells could therefore be more vulnerable to the suppression of either AKT1 or PDGFRα. Next, we measured the viability of Pten+/+ and Pten−/− MEFs in the presence of either an AKT inhibitor (MK-2206) or a PDGFR inhibitor (CP-673451). As shown in Fig. 8A, B, Pten−/− MEFs were more sensitive not only to MK-2206 treatment but also to CP-673451 intervention as compared with Pten+/+ MEFs. Furthermore, the combination of MK-2206 and CP-673451 demonstrated a stronger inhibitory effect on the proliferation of Pten-null MEFs than using any single agent alone, as shown by MTT and colony formation assays (Fig. 8C, D). The combination index (CI) was less than 1, implying a synergistic effect between MK-2206 and CP-673451 (Supplementary Fig. S5). Mechanically, MK-2206 in combination with CP-673451 treatment remarkably promoted the apoptosis of Pten−/− MEFs as demonstrated by increased cleavage of Caspase 3 (Fig. 8E). This increased apoptosis in response to the combined treatment with MK-2206 and CP-673451 was further confirmed by flow cytometry analyses (Fig. 8F). These findings prompted us to test the efficacy of this combinatorial strategy for the treatment of cancer in a preclinical animal model. U373 cells were injected s.c. into nude mice to establish a xenograft tumor model. As shown in Fig. 8G–I, the tumor growth was significantly inhibited by treatment with MK-2206 or CP-673451 alone, and MK-2206 in combination with CP-673451 exerted a dramatically better anti-tumor activity than either drug alone. Moreover, combination treatment did not significantly affect the body weight of mice (Fig. 8J).

## Discussion

Aberrant expression of PDGFRα has been identified in multiple types of human cancers, including medulloblastomas, papillary thyroid cancer, colorectal cancer, gliomas, liver cancer, and ovarian cancer^10,30–35^. Increasing evidence has demonstrated that PDGFRα plays a crucial role in cancer cell proliferation, metastasis, and the tumor microenvironment. For example, Lopez-Campistrous et al. reported that PDGFRα induced follicular cell dedifferentiation driving radioactive iodine therapy resistance in papillary thyroid cancer^34^. Cui and collaborators demonstrated that the upregulation of PDGFRα was critical for glioma tumorigenesis mediated by colon cancer-associated transcript 1 (CCAT1), a long non-coding RNA^36^. Zhu and colleagues found that overexpression of PDGFRα in the endothelial cells of hepatocellular carcinoma tissues was associated with microvascular invasion and was a predictor of a poor prognosis^37^. In this study, we showed that elevated PDGFRα expression accelerated the tumorigenic abilities of Pten-deficient cells and was associated with poor prognosis in multiple tumors. Our TCGA database analysis suggested that PDGFRα upregulation was positively correlated with the activation of the PI3K-AKT signaling pathway in human cancers. Moreover, we provide here mechanistic evidence that the activation of the AKT1-CREB signaling pathway was responsible for PDGFRα upregulation upon the loss of PTEN. Intriguingly, we found a positive feedback loop between AKT1 and PDGFRα upon PTEN loss, since the depletion of PDGFRα led to decreased AKT1 activity in Pten-null MEFs, while ectopic expression of PDGFRα resulted in activation of AKT1 in Pten+/+ MEFs (Supplementary Fig. S6). Therefore, PDGFRα may be a potential therapeutic target and prognostic biomarker for tumors with PTEN deficiency. These findings are of great significance since PTEN is one of the most frequently mutated tumor suppressor genes in cancer.

Since PDGFRα is crucial in promoting tumorigenesis, the upstream regulatory signaling pathways of PDGFRα expression have aroused increasing attention in recent years. In addition to the PTEN-AKT1 pathway we identified here, several other signaling cascades have also been shown to take part in tumorigenesis via modification of PDGFRα expression^23,31,38^. For example, Xie et al. reported that increased expression of PDGFRα was an important mechanism by which mutations in the hedgehog pathway cause basal cell carcinoma^31^. Afink et al. showed that interleukin-1β (IL-1β) signaling repressed PDGFRα expression in osteosarcoma MG-63 cells^38^. We have previously demonstrated that hyperactivated mTORC1 signaling suppresses the expression of PDGFRα, safeguarding against the development of malignant tumors in tuberous sclerosis complex (TSC), a benign tumor syndrome affecting multiple organs^11,23^. However, a detailed transcriptional regulation mechanism governing the expression of PDGFRα is far from being fully understood. Some transcription factors such as FOXO family members, Sp1, Phf14, and C/EBPβ have been implicated in the transcription of PDGFRα^38–41^, but only a few transcription factors have been shown to directly regulate PDGFRα in human cancer cells. For instance, Kikuchi and colleagues found that activated NFκB promoted the proliferation of Hep3B hepatoma cells via the upregulation of PDGFRα^9^. However, the inhibition of NFκB signaling in Pten-deficient cells by a pharmacological strategy had little effect on the expression of PDGFRα (Supplementary Fig. S7). Thus, the possibility that PDGFRα could be upregulated by NFκB in Pten-deficient cells could be ruled out. In addition, it has been reported that serum starvation leads to upregulation of PDGFRα through the inhibition of the AKT/FOXO pathway in neuroblastoma cells and MEFs^39^. However, it is well established that AKT1 phosphorylation of FOXO family members leads to their sequestration in the cytoplasm and inhibits their transcriptional activity^42^. In alignment with these findings, we found that serum deprivation led to the nuclear accumulation of FOXOs (FOXO1 and FOXO3a), as well as the upregulation of PDGFRα expression in control cells but no in myrAKT1 over-expressing MEFs (Supplementary Fig. S8). Thus, it seems that FOXOs were not involved in the transcriptional regulation of PDGFRα in Pten-deficient cells. Instead, in the current study, based on an analysis of mouse fibroblasts, human cancer cells, live tissues from Pten conditional knockout mice, and human invasive breast cancer tissues, we demonstrated that CREB, as a downstream target of the PTEN-AKT1 pathway, transcriptionally upregulates PDGFRα through directly binding to its promoter. Thus, PDGFRα is a downstream target of CREB at least in MEFs and cancer cells. Interestingly, in contrast to our study, Gu et al. reported that a loss of PTEN led to the activation of CREB in an AKT1-independent manner^43^. In that study, the authors showed that PTEN, as a nuclear phosphatase, dephosphorylated CREB and inhibited CREB-mediated gene transcription. Considering that cytoplasmic PTEN is primarily involved in regulating PI3K-AKT1 signaling, while nuclear PTEN exhibits lipid phosphatase-independent tumor-suppressive functions^44^, further studies are worthwhile to explore this dual mechanism by which PTEN regulates the activity of CREB in cancer cells.

Deregulation of the PI3K-AKT pathway is associated with tumorigenesis and disease progression in many types of cancer^2^. Thus, AKT inhibitors have been suggested to be potential drugs for the treatment of some advanced tumors, including breast cancer, renal cancer, and acute myelogenous leukemia (AML)^45^. To date, several clinical trials with MK-2206 have been carried out and some other trials are ongoing^46–48^. However, the therapeutic efficacy of MK-2206 has been shown to be rather limited. For example, Xing and colleagues reported that MK-2206 monotherapy had limited clinical activity in advanced breast cancer due to grade III side effects observed at the tolerable dose^48^. In another trial, in which the researcher treated 19 AML patients with MK-2206, only 1 response was observed, leading to early study termination^47^. The authors also showed that despite the use of MK-2206 at the maximum tolerated doses, only modest decreases in p-AKT Ser^473^ and limited inhibition of downstream targets were observed^47^. Reducing the cytotoxicity and increasing the treatment efficacy by a rational combination of different agents is a commonly used strategy for cancer therapies. For example, in a randomized phase II study, olaratumab, a human monoclonal antibody specific for PDGFRα, plus doxorubicin achieved a highly significant improvement in overall survival versus doxorubicin alone in patients with advanced or metastatic soft-tissue sarcoma^49^. PDGFRα belongs to a family of RTKs and is critical for PI3K-AKT activation^10^. In addition to demonstrating that loss of PTEN upregulated PDGFRα through activation of AKT1, we also identified that elevated PDGFRα led to feedback activation of AKT1 in PTEN deficiency cells. Thus, it would be interesting to investigate whether the combined administration of MK-2206 and a PDGFR inhibitor would achieve a better therapeutic effect than either agent alone in tumor treatment. Strikingly, we observed a synergistic anti-proliferative effect in vitro and in vivo after combined treatment with MK-2206 and CP-673451. As a mechanism, we found that CP-673451 enhanced MK-2206-induced apoptosis. Therefore, co-administration of MK-2206 and a PDGFR inhibitor may be an effective and novel strategy for the treatment of some PTEN-deficient cancers.

In summary, this study demonstrated that the loss of PTEN contributed to carcinogenesis through the upregulation of the AKT1-CREB-PDGFRα signaling cascade. These findings increase our understanding of tumor progression in PTEN-deficient cancers and the novel network regulating PDGFRα transcription. Importantly, the results of our work suggest that the combined administration of an AKT inhibitor with a PDGFR inhibitor may be a novel strategy for the treatment of some PTEN-deficient cancers.

## Supplementary information

Supplementary Table S1.

Supplementary Figure S1.

Supplementary Figure S2.

Supplementary Figure S3.

Supplementary Figure S4.

Supplementary Figure S5.

Supplementary Figure S6.

Supplementary Figure S7.

Supplementary Figure S8.

Supplementary Figure Legends
