# Supplementary material for: AKT1-CREB stimulation of PDGFRα expression is pivotal for PTEN deficient tumor development
Source: Cell Death Dis. 2021 Feb 10;12(2):172. doi: 10.1038/s41419-021-03433-0 (PMC7876135; doi:10.1038/s41419-021-03433-0)
Supplement: Supplementary file 1 — Supplementary Table S1. [file 41419_2021_3433_MOESM1_ESM.docx]

| Gene Symbol | Pten-/- vs Pten+/+ MEFs | Description |
| --- | --- | --- |
|  | (Fold change) |  |
| PDGFRα | 2.50448 | Up |
| PDGFRβ | -1.70831 | Down |
