# Supplementary figures and images for: AKT1-CREB stimulation of PDGFRα expression is pivotal for PTEN deficient tumor development
Source: Cell Death Dis. 2021 Feb 10;12(2):172. doi: 10.1038/s41419-021-03433-0 (PMC7876135; doi:10.1038/s41419-021-03433-0)

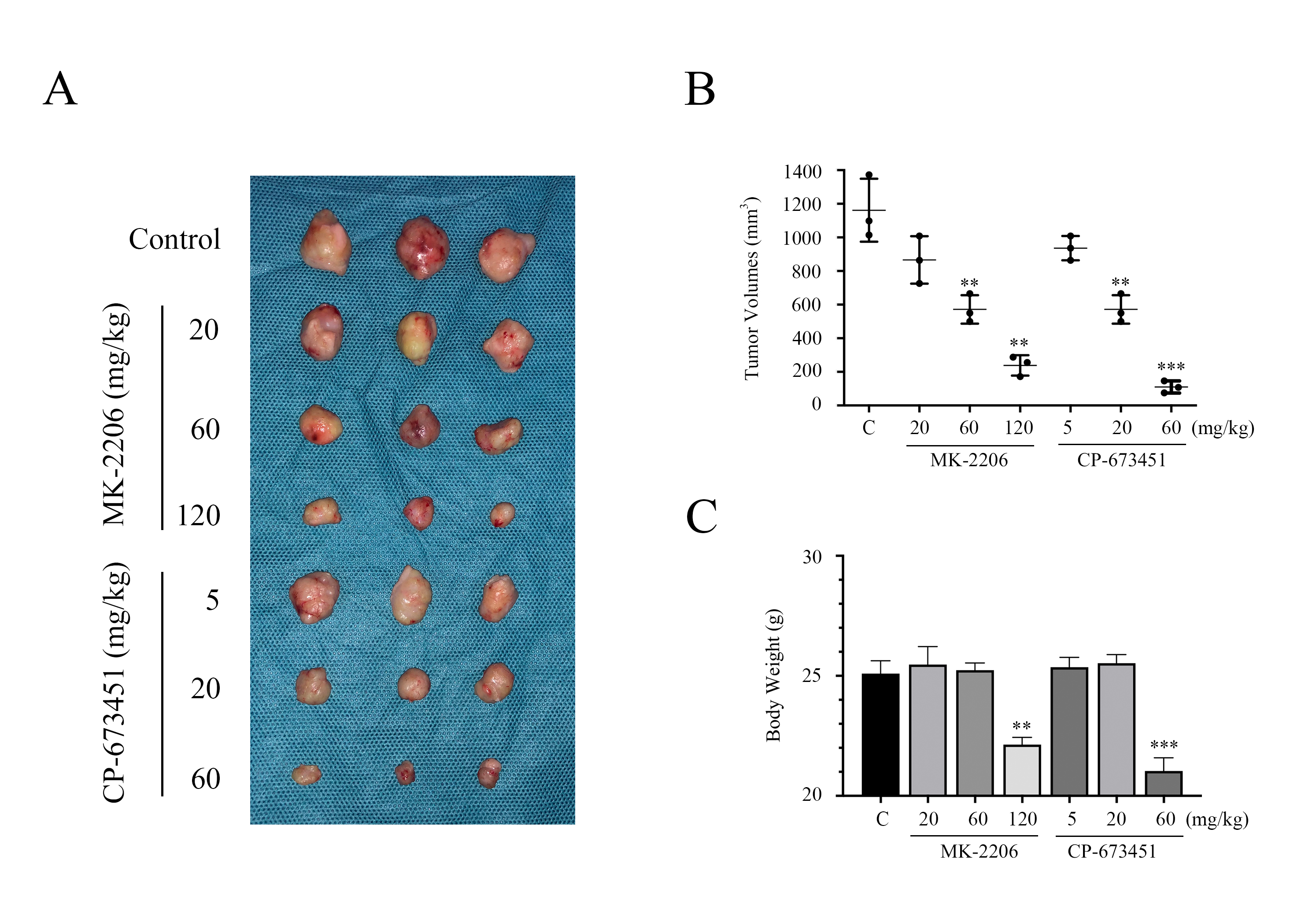

Supplement: Supplementary file 2 — Supplementary Figure S1. [file 41419_2021_3433_MOESM2_ESM.jpg]

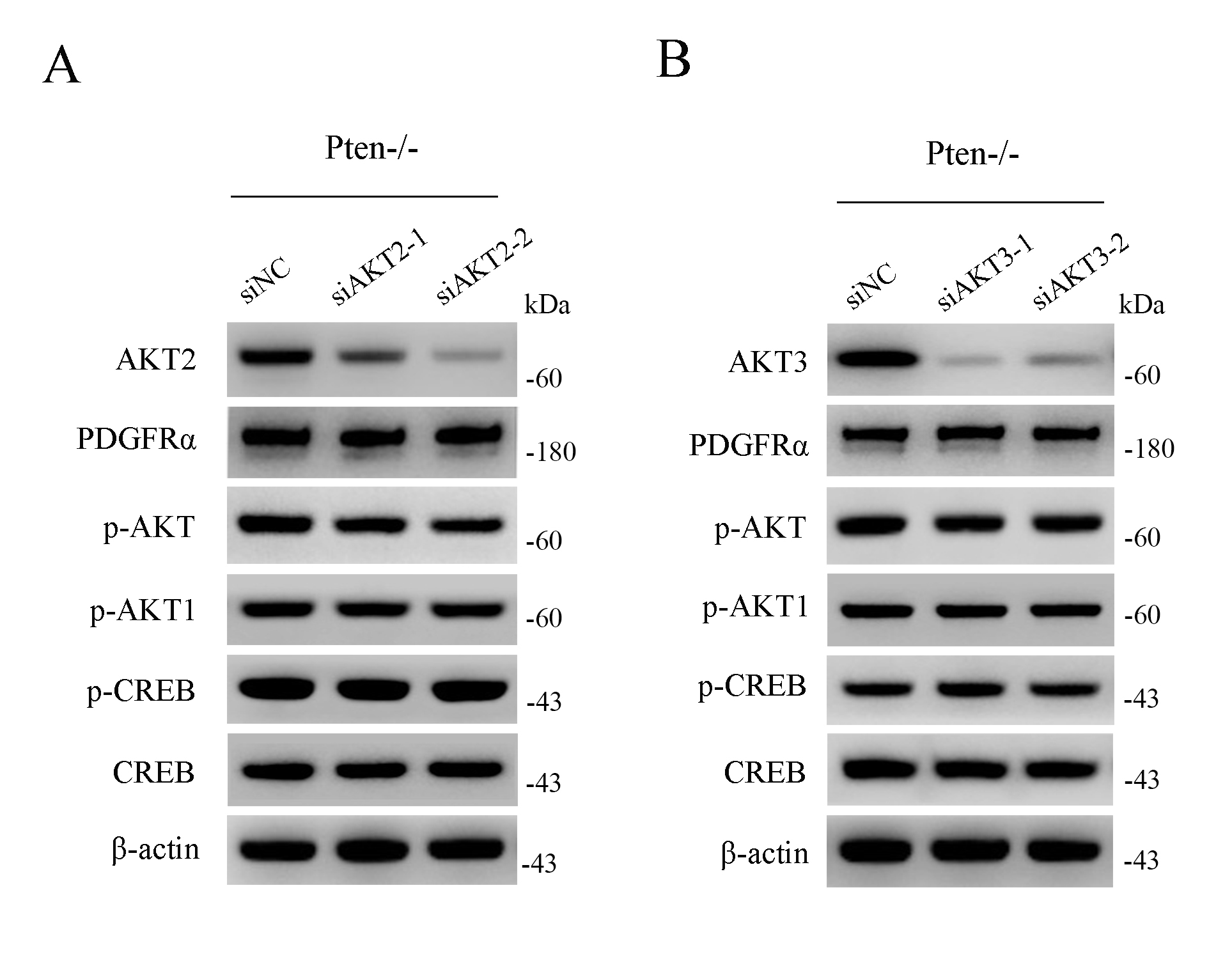

Supplement: Supplementary file 3 — Supplementary Figure S2. [file 41419_2021_3433_MOESM3_ESM.jpg]

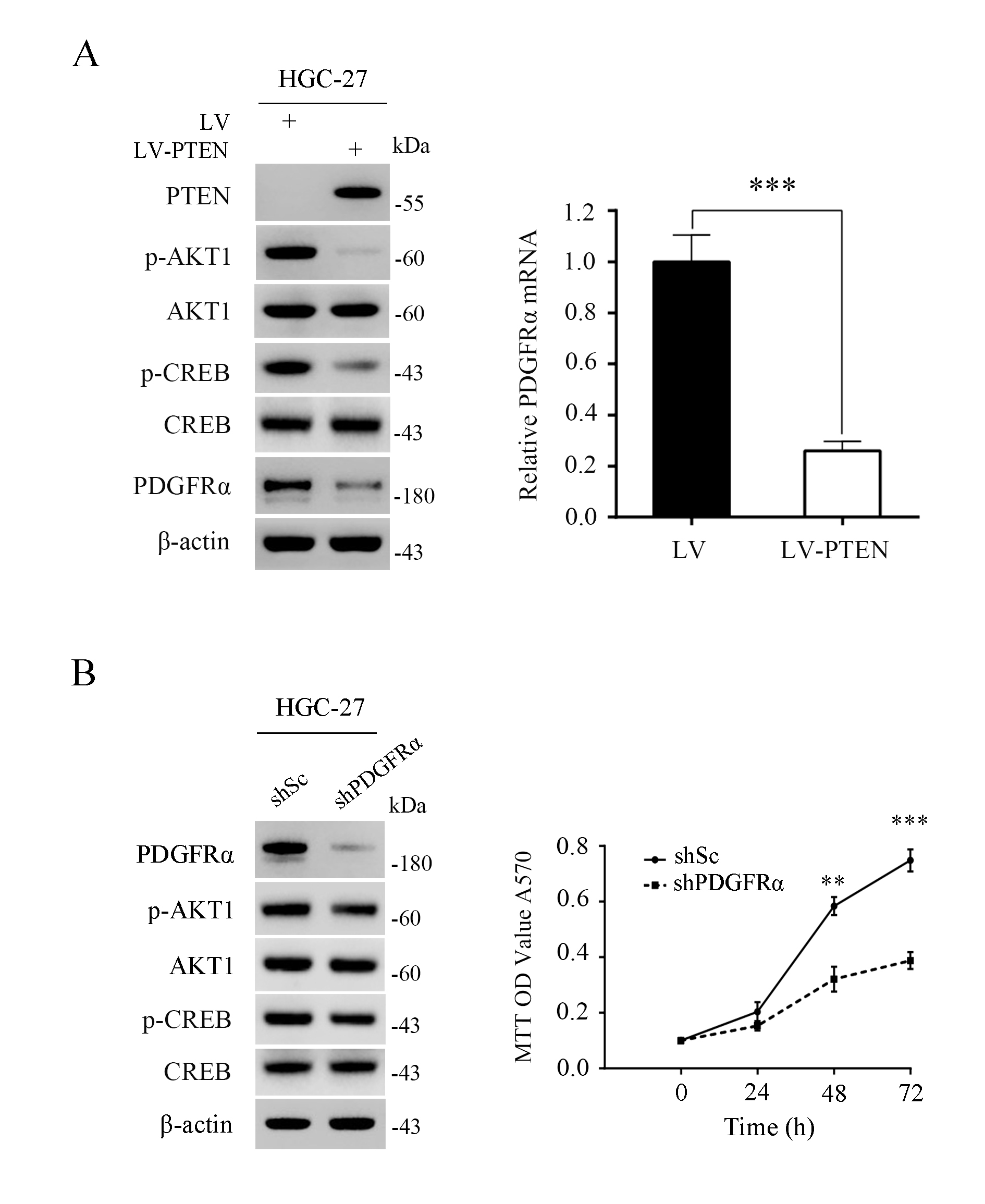

Supplement: Supplementary file 4 — Supplementary Figure S3. [file 41419_2021_3433_MOESM4_ESM.jpg]

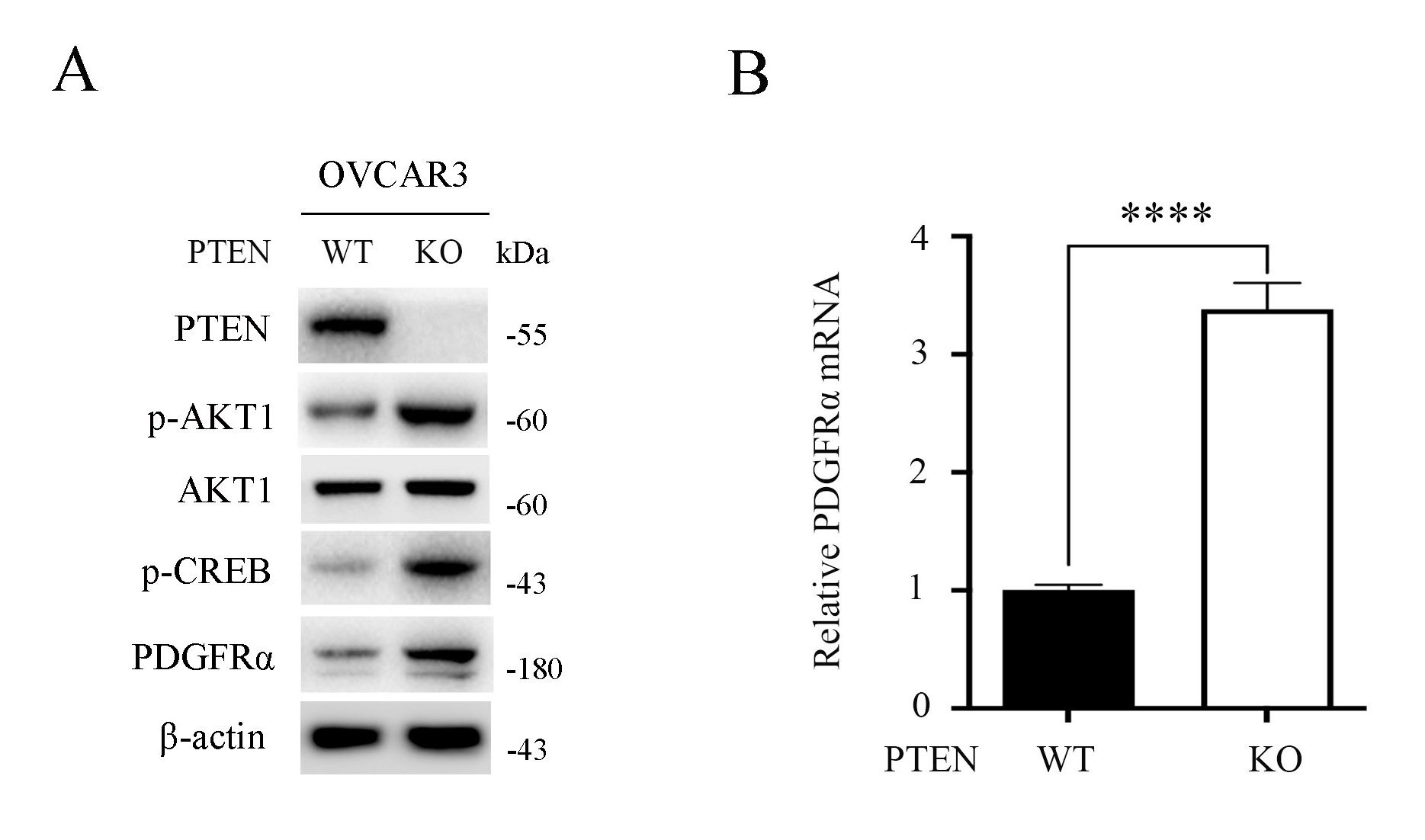

Supplement: Supplementary file 5 — Supplementary Figure S4. [file 41419_2021_3433_MOESM5_ESM.jpg]

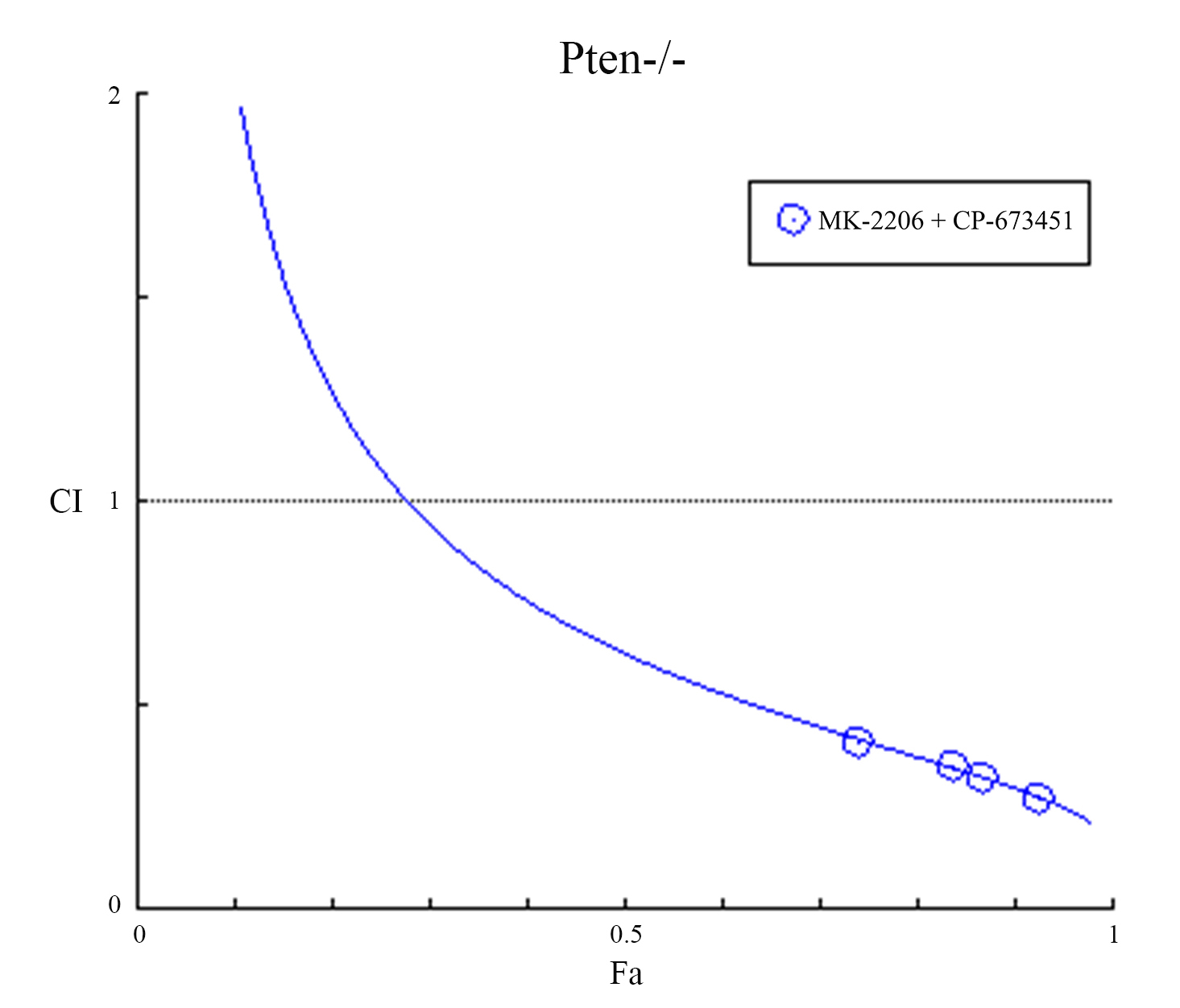

Supplement: Supplementary file 6 — Supplementary Figure S5. [file 41419_2021_3433_MOESM6_ESM.jpg]

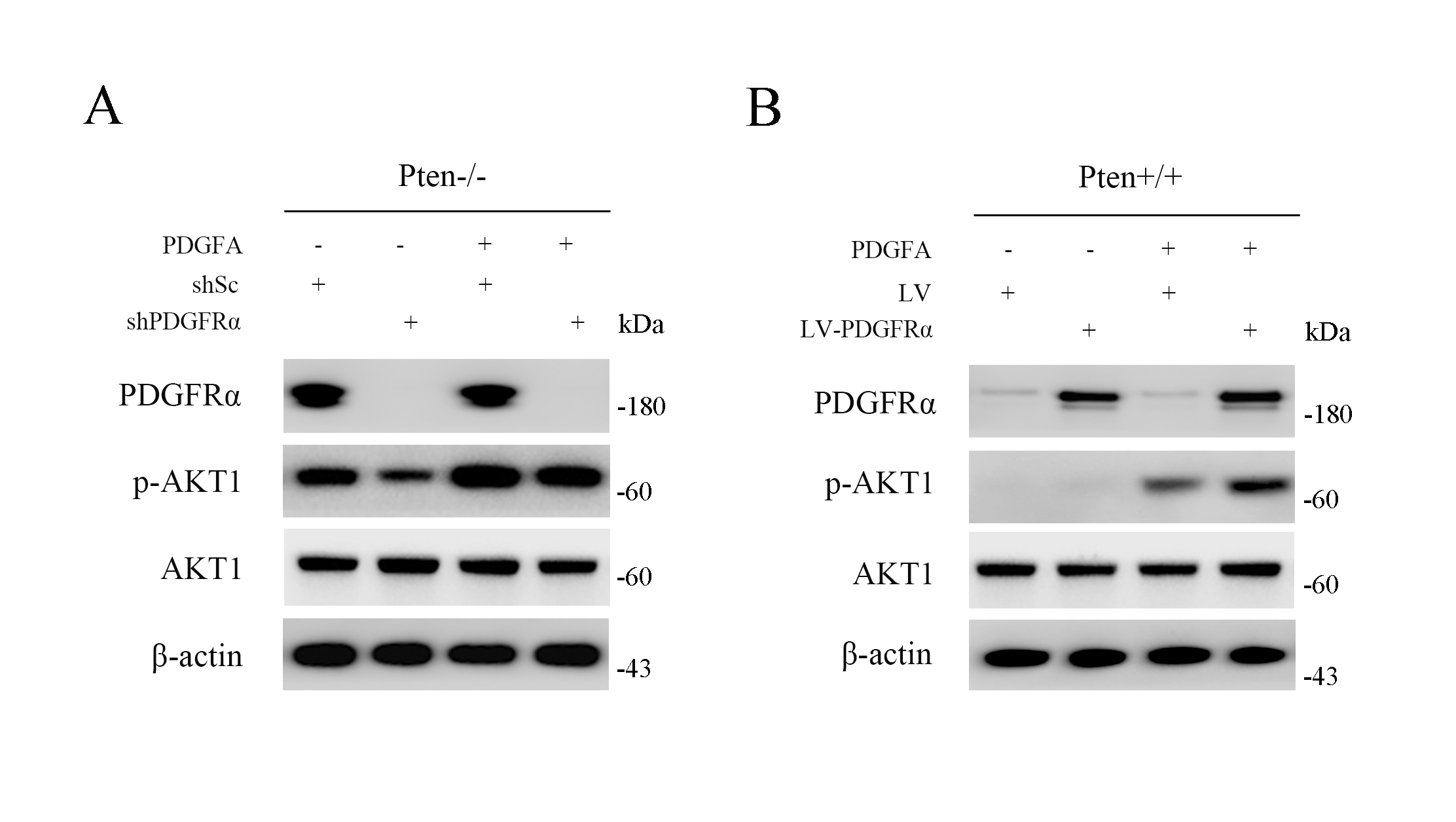

Supplement: Supplementary file 7 — Supplementary Figure S6. [file 41419_2021_3433_MOESM7_ESM.jpg]

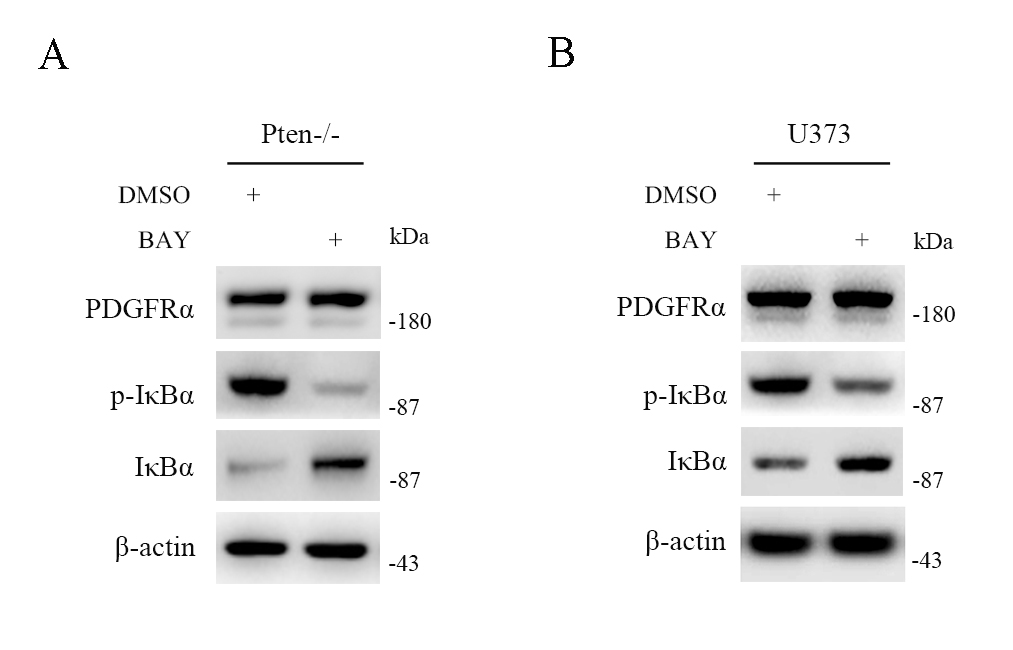

Supplement: Supplementary file 8 — Supplementary Figure S7. [file 41419_2021_3433_MOESM8_ESM.jpg]

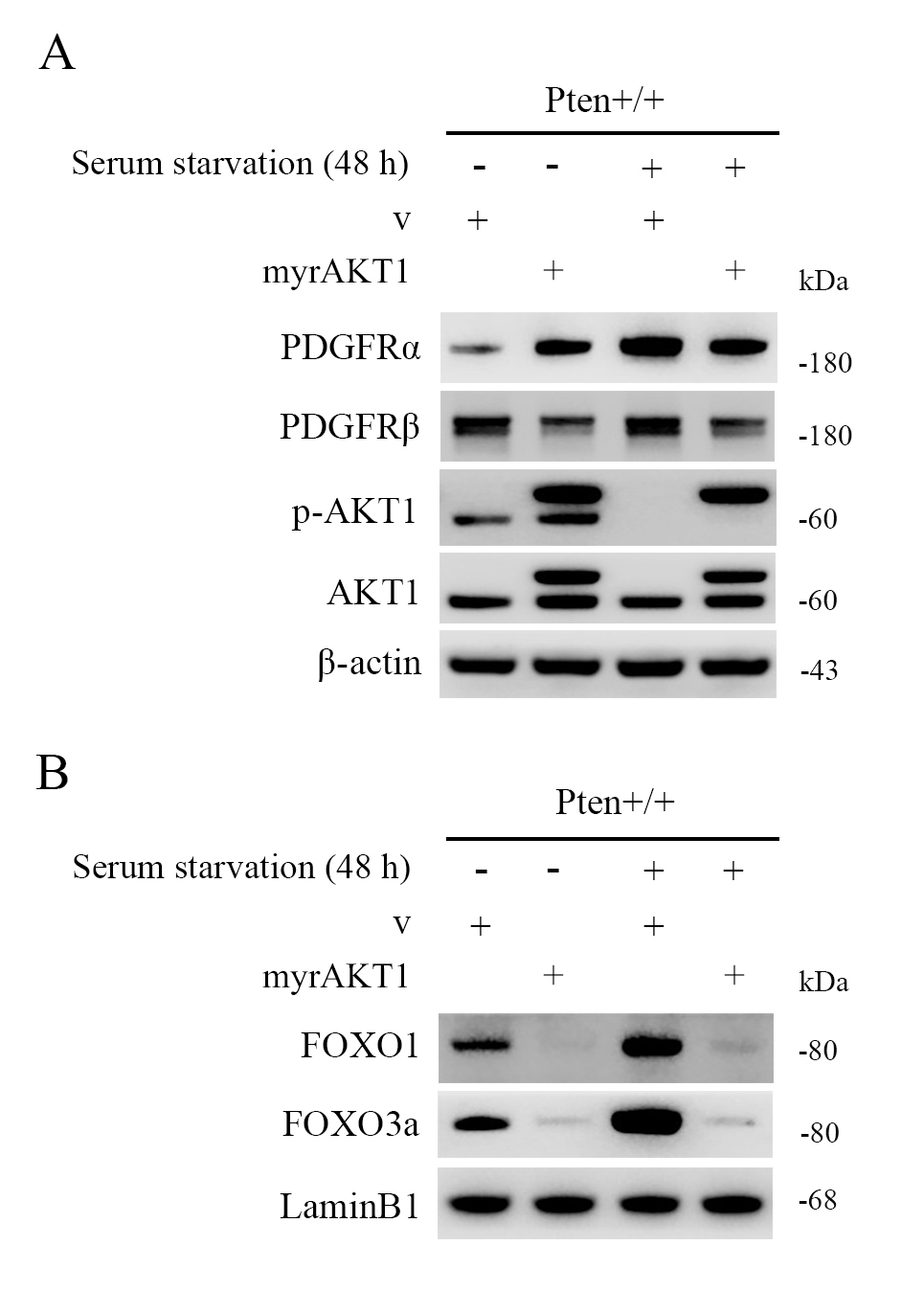

Supplement: Supplementary file 9 — Supplementary Figure S8. [file 41419_2021_3433_MOESM9_ESM.jpg]
