# Supplementary material for: AKT1-CREB stimulation of PDGFRα expression is pivotal for PTEN deficient tumor development
Source: Cell Death Dis. 2021 Feb 10;12(2):172. doi: 10.1038/s41419-021-03433-0 (PMC7876135; doi:10.1038/s41419-021-03433-0)
Supplement: Supplementary file 10 — Supplementary Figure Legends [file 41419_2021_3433_MOESM10_ESM.docx]

**Supplementary Materials**

**Supplementary Table S1.** PDGFRα mRNA is increased in Pten-null MEFs. mRNA abundance of PDGFRα and PDGFRβ in Pten+/+ and Pten-/- MEFs were measured using Affymetrix mouse genome 430 2.0 array.

**Supplementary Figure S1.** Preliminary experiments suggested that MK-2206 or CP-673451 suppresses the tumor growth of xenografted U373 cells. U373 cells (1 × 10^7^ cells/injection) were inoculated subcutaneously into the nude mice. After the tumors were detectable, the mice were randomly divided into seven groups (n=3) and treated with either vehicle solution alone (75% DMSO and 25% PBS), MK-2206 (20, 60, 120 mg/kg) or CP-673451 (5, 20, 60 mg/kg) by intraperitoneal injection, respectively (I.P., every two days, for a total of 10 injections). Two days after the tenth injection, the mice were sacrificed followed by dissection of the tumors. Dissected tumors (**A**). Tumor volumes (**B**). The body weight of mice (**C**). Error bars indicate mean ± SD of triplicate samples. ***P* < 0.01; ****P* < 0.001;

**Supplementary Figure S2.** Knockdown of AKT2 or AKT3 has little effect on the expression of PDGFRα. Pten-/- MEFs cells were transfected with control siRNAs (siNC), siRNAs against AKT2 (**A**), or AKT3 (**B**) for 48h, respectively. Cell lysates were subjected to immunoblotting.

**Supplementary Figure S3.** PDGFRα is critical for PTEN deficiency-induced cell proliferation in HGC-27 cells. **A** HGC-27 cells were transduced with LV-PTEN or LV lentiviruses. Cell lysates were subjected to immunoblotting (left panel), and qRT-PCR was performed to examine the expression of PDGFRα (right panel). **B** HGC-27 cells were stably expressing shPDGFRɑ or shSc. Cell lysates were analyzed for PDGFRα expression by the western blot (left panel). The proliferation of the indicated cells was examined using an MTT assay (right panel). Error bars indicate mean ± SD of triplicate samples. ***P* < 0.01; ****P* < 0.001.

**Supplementary Figure S4.** Knockout of PTEN led to the activation of AKT1-CREB -PDGFRα pathway in OVCAR3 cells. Pten wild-type (WT) or knockout (KO) OVCAR3 cells were subjected to western blot (**A**) and qRT-PCR (**B**) analyses. Error bars indicate mean ± SD of triplicate samples. *****P* < 0.0001.

**Supplementary Figure S5.** The combination treatment of MK-2206 and CP-673451 exerted a synergistically inhibitory effect on cell viability of Pten-/- MEFs. The combined effects of MK-2206 and CP-673451 were analyzed by CompuSyn software (<http://www.combosyn.com/index.html>). The Fa-CI plot of Pten-/- MEFs was showed. CI: combination index; Fa: fraction affected.

**Supplementary Figure S6.** PDGFRα is critical for AKT1 activation. Pten-/- MEFs transduced with shPDGFRα or shSc lentiviruses (**A**). Pten+/+ MEFs transduced with LV-PDGFRα or LV lentiviruses (**B**). Cells were starved in DMEM for 12 h, followed by stimulation with PDGFA (10 ng/ml) for 15 min, and then cell lysates were harvested and subjected to immunoblotting with the indicated antibodies.

**Supplementary Figure S7.** Inhibition of NFκB did not affect the expression of PDGFRα. Pten-/- MEFs (**A**) and U373 (**B**) cells were treated with or without 10 μM BAY 11-7082 (BAY) for 24 h. The proteins were detected by immunoblotting.

**Supplementary Figure S8.** Serum starvation activated FOXOs-PDGFRα pathway in the control cells but not in myrAKT1 overexpressing cells. myrAKT1 overexpressing Pten+/+ MEFs and the control cells were cultured in complete medium or serum starved medium for 48 h. The whole cell lysates were subjected to immunoblotting with the indicated antibodies (**A**). The nuclear extracts were subjected to western blot analysis using anti-FOXO1 and anti-FOXO3a antibodies (**B**).
